# A signal recognition particle-related joint model of LASSO regression, SVM-RFE and artificial neural network for the diagnosis of systemic sclerosis-associated pulmonary hypertension

**DOI:** 10.3389/fgene.2022.1078200

**Published:** 2022-11-28

**Authors:** Jingxi Xu, Chaoyang Liang, Jiangtao Li

**Affiliations:** ^1^ North Sichuan Medical College, Nanchong, China; ^2^ Department of Rheumatology and Immunology, The First People’s Hospital of Yibin, Yibin, China

**Keywords:** systemic sclerosis-associated pulmonary hypertension, signal recognition particle, machine learning, artificial neural network, diagnostic model

## Abstract

**Background:** Systemic sclerosis-associated pulmonary hypertension (SSc-PH) is one of the most common causes of death in patients with systemic sclerosis (SSc). The complexity of SSc-PH and the heterogeneity of clinical features in SSc-PH patients contribute to the difficulty of diagnosis. Therefore, there is a pressing need to develop and optimize models for the diagnosis of SSc-PH. Signal recognition particle (SRP) deficiency has been found to promote the progression of multiple cancers, but the relationship between SRP and SSc-PH has not been explored.

**Methods:** First, we obtained the GSE19617 and GSE33463 datasets from the Gene Expression Omnibus (GEO) database as the training set, GSE22356 as the test set, and the SRP-related gene set from the MSigDB database. Next, we identified differentially expressed SRP-related genes (DE-SRPGs) and performed unsupervised clustering and gene enrichment analyses. Then, we used least absolute shrinkage and selection operator (LASSO) regression and support vector machine-recursive feature elimination (SVM-RFE) to identify SRP-related diagnostic genes (SRP-DGs). We constructed an SRP scoring system and a nomogram model based on the SRP-DGs and established an artificial neural network (ANN) for diagnosis. We used receiver operating characteristic (ROC) curves to identify the SRP-related signature in the training and test sets. Finally, we analyzed immune features, signaling pathways, and drugs associated with SRP and investigated SRP-DGs’ functions using single gene batch correlation analysis-based GSEA.

**Results:** We obtained 30 DE-SRPGs and found that they were enriched in functions and pathways such as “protein targeting to ER,” “cytosolic ribosome,” and “coronavirus disease—COVID-19”. Subsequently, we identified seven SRP-DGs whose expression levels and diagnostic efficacy were validated in the test set. As one signature, the area under the ROC curve (AUC) values for seven SRP-DGs were 0.769 and 1.000 in the training and test sets, respectively. Predictions made using the nomogram model are likely beneficial for SSc-PH patients. The AUC values of the ANN were 0.999 and 0.860 in the training and test sets, respectively. Finally, we discovered that some immune cells and pathways, such as activated dendritic cells, complement activation, and heme metabolism, were significantly associated with SRP-DGs and identified ten drugs targeting SRP-DGs.

**Conclusion:** We constructed a reliable SRP-related ANN model for the diagnosis of SSc-PH and investigated the possible role of SRP in the etiopathogenesis of SSc-PH by bioinformatics methods to provide a basis for precision and personalized medicine.

## Introduction

Systemic sclerosis (SSc) is a type of connective tissue disease (CTD). There are three main characteristics of SSc: inflammation, fibrosis, and vasculopathy (Denton and Khanna, 2017). In the early stage, the pathological process of SSc is predominantly inflammatory and may manifest as swollen fingers, inflammatory skin disease, and musculoskeletal inflammation (Sticherling, 2019). In the advanced stage, the pathological process of SSc is dominated by fibrosis and vasculopathy, which can manifest as lung fibrosis, cardiac fibrosis, pulmonary hypertension (PH), and even scleroderma renal crisis (Asano, 2020). Among the major complications of SSc, PH significantly impacts the mortality of SSc patients (Xiong et al., 2022). Some studies have shown that the 3-year survival rate for patients with systemic sclerosis-associated pulmonary hypertension (SSc-PH) is between 31% and 52%, while the 5-year survival rate is <50% (Foocharoen et al., 2011; Humbert et al., 2011; Lefèvre et al., 2013). Therefore, it is indispensable to develop methods to predict the risk of complications from PH in SSc patients.

However, SSc-PH is a rare disease that develops insidiously, and the early symptoms of SSc-PH, such as fatigue and dyspnea, are nonspecific, thus making diagnoses difficult (Yorke et al., 2014). Researchers have now made progress in developing methods to screen for SSc-PH. The guidelines of the European Society of Cardiology and European Respiratory Society have identified several methods to screen for PH, such as electrocardiography, cardiopulmonary exercise testing, Doppler transthoracic echocardiography, and pulmonary function tests, which are applicable to SSc patients (Galiè et al., 2016). Meanwhile, several algorithms have been developed to screen for SSc-PH. The DETECT algorithm is a noninvasive, two-step predictive algorithm that can be used to evaluate the risk of PH complications in adult SSc patients (Coghlan et al., 2014). It was demonstrated that the sensitivity, specificity, positive predictive value, and negative predictive value of SSc-PH detection when using the DETECT algorithm were 100%, 42.9%, 68.6%, and 100%, respectively (Guillén-Del Castillo et al., 2017). Meanwhile, the Australian Scleroderma Interest Group developed the ASIG algorithm for screening SSc-PH based on NT-proBNP levels and lung function test results, which yielded sensitivity, specificity, positive predictive value, and negative predictive value of 94.1%, 54.5%, 61.5%, and 92.3%, respectively (Thakkar et al., 2013). Although both algorithms effectively screen SSc-PH, their specificities are suboptimal, and further cost-effective evaluations are needed (Kiely et al., 2019).

Several circulating proteins, such as NT-proBNP, endothelin, and vascular endothelial growth factor, have been determined to be biomarkers of SSc-PH (Hickey et al., 2018). Moreover, several microRNAs, such as miR-424, miR-4632, and miR-193b, showed potential as biomarkers of pulmonary vascular remodeling in SSc patients (Odler et al., 2018). In addition, Bauer et al. (2021) identified a proteomic biomarker signature by using machine learning that could improve the specificity of the DETECT algorithm. Zheng et al. (2020) and Tu et al. (2022) identified hub genes of SSc-PH by multiple bioinformatic methods based on microarray data mining. Lui et al. (2022) constructed and compared the performance features of three SSc-PH prediction models using pulmonary function tests, electrocardiography, and imaging data. However, there is a lack of research on constructing diagnostic models for SSc-PH by machine learning based on microarray data. According to our literature review, no studies predicting SSc-PH risk based on artificial neural network (ANN) models have been reported.

The signal recognition particle (SRP) is a ribonucleoprotein formed by 7SL RNA and six protein subunits (SRP9, SRP14, SRP19, SRP54, SRP68, and SRP72 proteins) (Pool, 2022). The main function of SRP is to cotranslationally target many secretory and membrane proteins to the endoplasmic reticulum (ER) (Kellogg et al., 2022). Studies have shown that SRP depletion leads to protein mislocalization to mitochondria, further leading to mitochondrial dysfunction and decreased cell survival (Karamyshev et al., 2020; Hsieh and Shan, 2021). In addition, SRP depletion also leads to pathological activation of the Regulation of Aberrant Protein Production (RAPP), a process implicated in various diseases, including hepatocellular cancer, colorectal cancer, and Alzheimer’s disease (Kellogg et al., 2022). However, whether SRP depletion functions in the progression of SSc evolving into SSc-PH has not been explored.

In this study, we attempted to construct a novel SRP-related ANN model for the early diagnosis and assessment of SSc-PH and to investigate the role of SRP-related genes in the pathogenesis of SSc-PH. We first revealed two SRP expression patterns in SSc-PH and evaluated the signal transduction and immune characteristics in different SRP expression patterns. Next, we identified SRP-related diagnostic genes (SRP-DGs) for SSc-PH using machine learning algorithms and validated the diagnostic efficacy of these SRP-DGs in the test set. Subsequently, we constructed an SRP scoring system called SRPscore, evaluated the relationship between SRPscore and SRP expression patterns and immune characteristics, and constructed a nomogram model. Finally, we constructed a novel ANN model for SSc-PH diagnosis and validated the accuracy of the ANN model in the test set. Moreover, we also revealed the associations between SRP-DGs with immune signature and SSc-PH-related pathways, explored SRP-DGs’ functions using single gene batch correlation analysis-based GSEA, and screened for drugs that may target and regulate SRP-DGs.

## Materials and methods

### Data downloading

We downloaded the datasets from the GEO database, and those that met the following criteria were included in our study: 1) Studies including both peripheral blood mononuclear cell (PBMC) samples from SSc-PH patients and PBMC samples from SSc patients without pulmonary hypertension. 2) Studies whose data and platform information were complete. Three datasets (GSE19617, GSE3346, and GSE22356) were included in our study. Specifically, GSE19617 contains 17 PBMC samples from SSc-PH patients and 25 PBMC samples from SSc patients without pulmonary hypertension, GSE33463 contains 42 PBMC samples from SSc-PH patients and 19 PBMC samples from SSc patients without pulmonary hypertension, and GSE22356 contains 10 PBMC samples from SSc-PH patients and 10 PBMC samples from SSc patients without pulmonary hypertension. Table 1 presents information about the datasets utilized in this study.

**TABLE 1:** The information about the datasets utilized in this study.

GEO accession	Platform	SSc	SSc-PH	Set
GSE19617	GPL6480	25	17	Training
GSE33463	GPL6947	19	42	Training
GSE22356	GPL570	10	10	Test

### Data processing

First, the array probes in the three datasets were transformed into matched gene symbols based on the platform annotation information. Then, to decrease the sample selection bias caused by the different distributions in the training and test sets, it was necessary to make the ratio of the sample size of the treatment group to the sample size of the control group in the training set close to the ratio of the sample size of the treatment group to the sample size of the control group in the test set, so we merged the mRNA expression data in GSE19617 and GSE33463 as the training set and selected GSE22356 as the test set (Bickel et al., 2007). GSE19617 was based on the GPL6480 platform, in which the mRNA expression data had been normalized by the researchers; GSE33463 and GSE22356 were based on the GPL6947 and GPL570 platforms, respectively, in which the mRNA expression data were not normalized (Pendergrass et al., 2010). We used the R package, “limma,” to normalize the mRNA expression data in the GSE33463 and GSE22356 datasets. Subsequently, to remove the batch effect caused by different platforms and different normalization methods, after studying the literature, we found that “ComBat” in the R package, “sva,” can efficiently remove the batch effect among data generated by different laboratories on account of different platforms (Johnson et al., 2007; Thillaiyampalam et al., 2017; Tang et al., 2021). Therefore, we merged the normalized mRNA expression data from GSE19617 and GSE33463 and used “ComBat” in the R package, “sva,” to remove the batch effect (Leek et al., 2012). Through our literature review, we found that among the dimensionality reduction algorithms, both t-distributed stochastic neighbor embedding (tSNE) and uniform manifold approximation and projection (UMAP) can effectively analyze sample-to-sample heterogeneity and detect batch effects (Yang Y. et al., 2021; Xiang et al., 2021). Therefore, we evaluated the efficacy of removing the batch effect by tSNE and UMAP. In addition, we also used these two methods to analyze the difficulty in distinguishing SSc-PH patients from SSc patients without pulmonary hypertension. SangerBox was used to visualize the results (Shen et al., 2022).

### Differentially expressed SRP-related genes

We obtained 113 SRP-related genes from the “REACTOME_SRP_DEPENDENT_COTRANSLATIONAL_PROTEIN_TARGETING_TO_MEMBRANE.v7.5.1” gene set in the MSigDB database. We used SSc-PH patients and SSc patients without pulmonary hypertension as the treatment group and control group, respectively, and used the R package, “limma,” with a *p*-value < 0.05 as the criterion to filter out the differentially expressed SRP-related genes (DE-SRPGs) between the treatment and control groups in the training set (Ritchie et al., 2015). The *p* values were calculated using the Wilcoxon rank sum test. In addition, we verified the expression patterns of the DE-SRPGs in the test set.

### Unsupervised clustering

We performed an unsupervised clustering analysis of the SSc-PH patients in the training set based on the DE-SRPGs using the R package, “ConsensusClusterPlus,” (Wilkerson and Hayes, 2010). According to the clustering effect, the clustering stability was higher when *k* = 2. Therefore, we categorized the SSc-PH patients from the training set into two SRP clusters (SRPcluster A and SRPcluster B) based on the unsupervised clustering results. To further evaluate the relationships among SRPcluster A, SRPcluster B, and the control group, we performed dimensionality reduction of the training set using tSNE and UMAP based on the expression of DE-SRPGs.

### Pathway analysis

To explore the differences in signal transduction between SRPcluster A and SRPcluster B, we downloaded the file, “c2.cp.kegg.v2022.1.Hs.symbols.gmt,” from the MSigDB database for gene set enrichment analysis (GSEA). We performed GSEA using the R package, “clusterProfiler,” and the statistical significance was set to an adjusted *p*-value of <0.05 (Yu et al., 2012; Wu et al., 2021). Then, GO annotation and KEGG pathway enrichment analysis of the DE-SRPGs were performed using the R package “clusterProfiler.” Significantly enriched signaling pathways were identified using a *p*-value <0.05 as the criterion. The results were visualized using the R packages, “ggplot2″ and “ComplexHeatmap” (Gu et al., 2016). A single-sample gene set enrichment analysis (ssGSEA) of 29 immune gene sets was performed using the R package, “GSVA” (Hänzelmann et al., 2013). The enrichment scores of 29 immune gene sets in each sample were calculated. Similarly, we obtained 14 SSc-PH-related pathway gene sets from the MSigDB database and performed ssGSEA on 14 SSc-PH-related pathway gene sets. Then, we compared the normalized ssGSEA scores of the treatment and control groups and the normalized ssGSEA scores of SRPcluster A and SRPcluster B. The metagenes of 14 SSc-PH-related pathways are shown in Supplementary Table S1.

### Identification of SRP-related diagnostic genes using LASSO regression and SVM-RFE

For DE-SRPGs, we performed LASSO (least absolute shrinkage and selection operator) regression and SVM-RFE (support vector machine-recursive feature elimination) to identify the optimal signal recognition particle-related diagnostic genes (SRP-DGs) for SSc-PH. For both LASSO regression and SVM-RFE, the seed setting was 123. LASSO regression analysis was performed using the R package, “glmnet,” and SVM-RFE using the R package, “e1071” (Friedman et al., 2010). The SRP-related markers that were identified by the two algorithms were intersected, the intersecting genes were identified as the SRP-DGs, and the accuracy of the SRP-DGs for diagnosis in the training and test sets was evaluated using the receiver operating characteristic curve (ROC). We also compared the expression levels of SRP-DGs in SRPcluster A, SRPcluster B, and the control group.

### Construction of the SRP scoring system

To further analyze the diagnostic efficacy of the SRP-DGs, we constructed an SRP scoring system based on the SRP-DGs. We referred to the method of previous studies and performed a principal component analysis based on the expression levels of SRP-DGs and used principal component 1 and principal component 2 as feature scores (Sotiriou et al., 2006; Zhang et al., 2020; Zhang et al., 2022). The formula for calculating the SRPscore is:

$$SRPscore=\sum \left(PC1i+PC2i\right)$$

In the formula, “i” represents the expressions of SRP-DGs. Then, we compared the SRPscore values of the control and treatment groups. Subsequently, we categorized the samples with SRPscore >0 as the high SRPscore group and those with SRPscore ≤0 as the low SRPscore group and analyzed the correlation between SRPscore and SRPcluster. Finally, we compared the normalized ssGSEA scores of 29 immune gene sets in the high SRPscore group with the low SRPscore group. We used ROC to evaluate the accuracy of the SRPscore values for diagnosis in the training and test sets. We then compared SRPscore values in SRPcluster A, SRPcluster B, and the control group.

### Construction of a nomogram model

To predict the risk of SSc-PH, we constructed a nomogram based on the expression levels of the SRP-DGs using the R package, “rms.” We then plotted a calibration curve to determine the extent to which the predicted values corresponded to reality. We carried out a decision curve analysis (DCA) and plotted a clinical impact curve to determine whether clinical decisions based on the nomogram model were beneficial to patients.

### Construction and verification of the ANN model

We constructed an ANN model using the SRP-DGs. After the gene expression data were normalized using the min-max normalization method, the seed was set to 123. An ANN model was constructed using the R package, “neuralnet.” The ANN consists of three layers: 1) Input layer, which includes the gene expressions of the seven SRP-DGs normalized by the min-max method; 2) hidden layer, which includes the gene expressions of the seven SRP-DGs normalized by the min-max method and the weights of the seven SRP-DGs; 3) output layer, which represents the results of determining whether the samples belong to the control group or treatment group. The number of neurons in the hidden layer should be two-thirds of the number of neurons in the input layer plus two-thirds of the number of neurons in the output layer, and should be in the range between the number of neurons in the input layer and the number of neurons in the output layer (Sheela and Deepa, 2013). Therefore, we set the number of neurons in the hidden layer to six and used the ROC to evaluate the predictive performance of the ANN in the training and test sets.

### Correlation of SRP-DGs with immune characteristics and SSc-PH-related pathways

To assess the correlations between SRP-DGs with immune features and SSc-PH-related pathways, we calculated Spearman’s rank correlation coefficients and *p* values of the SRP-DGs with normalized ssGSEA scores of 29 immune gene sets and 14 SSc-PH-related pathways, which were visualized using the R package, “ggplot2.”

### GSEA based on single gene batch correlation analysis

To further explore SRP-DGs’ functions, we performed GSEA based on single gene batch correlation analysis for each SRP-DG. The idea is to calculate Spearman’s rank correlation coefficients and *p* values for all genes in the training set with a single gene and to perform GSEA for genes that are significantly positively and negatively correlated with a single gene, respectively, thus simulating the possible involvement of a single gene in activation and suppression of signaling pathways. See Supplementary Table S2 for the code.

### Screening of drugs associated with SRP-DGs

Using the Enrichr platform (https://maayanlab.cloud/Enrichr/), we entered the gene names of the SRP-DGs and screened for drugs associated with the SRP-DGs based on the DSigDB database in the “Diseases/Drugs” module with a criterion of *p*-value < 0.05 (Kuleshov et al., 2016).

## Results

### Gene expression data processing

**FIGURE 1:**
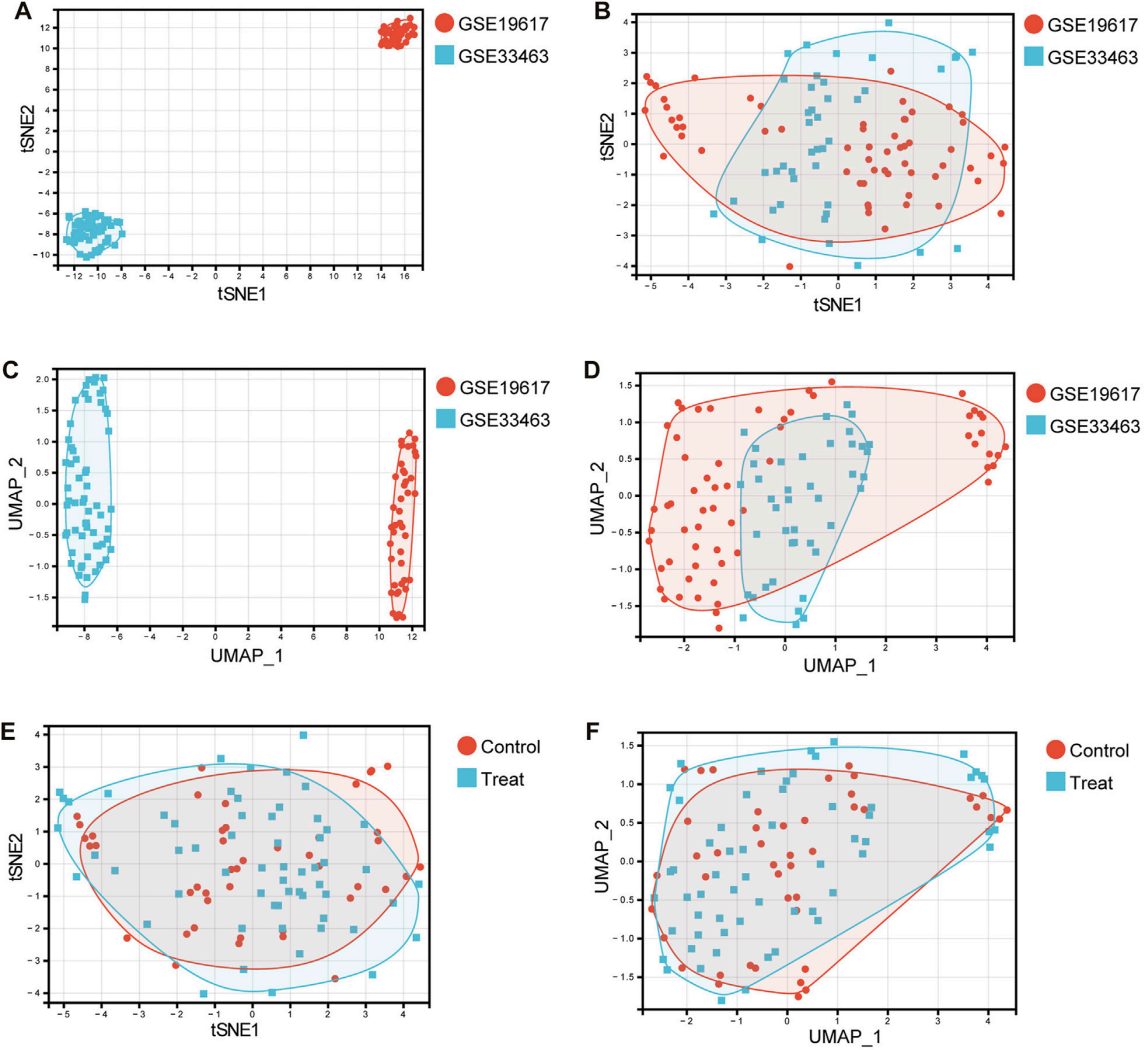
Dimensionality reduction using tSNE and UMAP. **(A)** The tSNE plot before removal of the batch effect. Red dots represent samples in the GSE19617 dataset, and blue squares represent samples in the GSE33463 dataset. **(B)** The tSNE plot after removal of the batch effect. Red dots represent samples in the GSE19617 dataset, and blue squares represent samples in the GSE33463 dataset. **(C)** The UMAP plot before removal of the batch effect. Red dots represent samples in the GSE19617 dataset, and blue squares represent samples in the GSE33463 dataset. **(D)** The UMAP plot after removal of the batch effect. Red dots represent samples in the GSE19617 dataset, and blue squares represent samples in the GSE33463 dataset. **(E)** The tSNE plot of the control and treatment group samples. Red dots represent control group samples, and blue squares represent treatment group samples. **(F)** The UMAP plot of the control and treatment group samples. Red dots represent control group samples, and blue squares represent treatment group samples.

Data heterogeneity and batch effects exist between datasets from different studies, which will adversely affect subsequent analyses if not correctly handled. Figures 1A,C show the tSNE plot and UMAP plot of the samples from the GSE19617 and GSE33463 datasets. As shown in Figures 1A,C, there was a clear difference between GSE19617 and GSE33463. Therefore, we must remove the batch effect before proceeding with the analysis. We used the “ComBat” function from the R package, “sva” to remove the batch effect. In the “sva” package, the “sva” function can be used for variable estimation, and the “ComBat” function removes batch effects, thereby reducing dependencies, stabilizing error rate estimates, and improving the reproducibility of the analysis (Leek et al., 2012). Figures 1B,D show the tSNE and UMAP plots for the samples from GSE19617 and GSE33463 after we removed the batch effect using the ComBat function. The results showed that the batch effect between GSE19617 and GSE33463 was removed and the data could then be used for subsequent analyses. In addition, tSNE (Figure 1E) and UMAP (Figure 1F) for the control group (SSc patients without pulmonary hypertension) and treatment group (SSc-PH patients) revealed no significant differences between the control and treatment groups, suggesting diagnostic difficulties.

### Differential analysis of PBMC samples from SSc-PH patients and SSc patients without pulmonary hypertension

**FIGURE 2:**
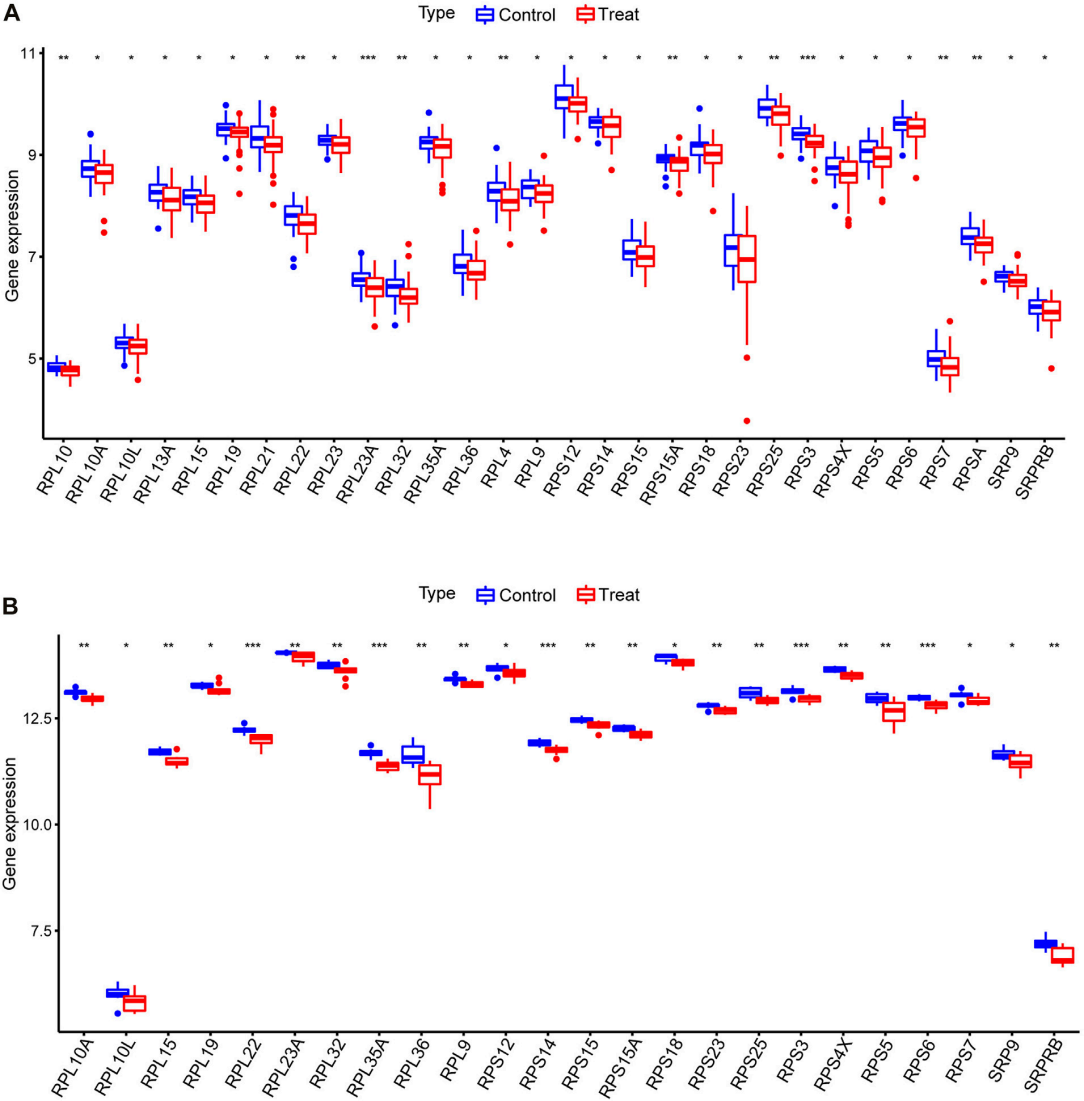
Differential analysis of DE-SRPGs. **(A)** Box plot of 30 DE-SRPGs in the training set. **(B)** Box plot of the 24 DE-SRPGs (excluding the six missing DE-SRPGs) in the test set. Red denotes the treatment group, and blue denotes the control group. *, *p* < 0.05; **, *p* < 0.01; ***, *p* < 0.001.

We performed a differential analysis of 113 SRP-related genes present in the PBMC samples from SSc-PH patients *versus* SSc patients without pulmonary hypertension in the training set. The results showed that 30 differentially expressed SRP-related genes (DE-SRPGs) were identified using *p* < 0.05 as the criterion (Supplementary Table S3). Figure 2A is a box plot of the 30 DE-SRPGs. Notably, all 30 DE-SRPGs were downregulated in SSc-PH. Subsequently, we verified the expression patterns of the DE-SRPGs in the test set. Due to platform differences, the expressions of RPL10, RPL13A, RPL21, RPL23, RPL4, and RPSA were missing in the test set (GSE22356) among the 30 DE-SRPGs. In the test set, all 24 DE-SRPGs were also significantly downregulated in SSc-PH, except for 6 DE-SRPGs that were missing due to platform differences (Figure 2B, Supplementary Table S3). This suggests that SRP-related dysfunctions and defects may occur in the pathogenesis of SSc-PH. For further investigation, we clustered SSc-PH patients based on the DE-SRPGs and performed gene enrichment analysis.

### Identification of two SRP clusters based on the expression patterns of DE-SRPGs

**FIGURE 3:**
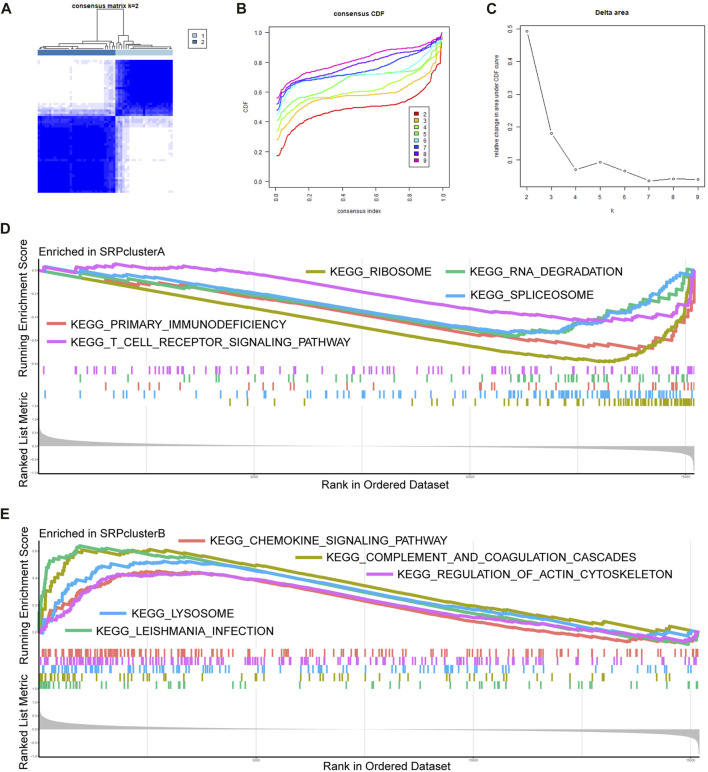
Unsupervised clustering and GSEA. **(A)** Consensus matrix. **(B)** A CDF graph illustrating the clustering according to DE-SRPGs. **(C)** Variation of the area under the CDF curve for *k* = 2–9. **(D)** The top five enriched KEGG pathways in SRPcluster A.**(E)** The five most significantly enriched KEGG pathways in SRPcluster B. Adjusted *p*-value < 0.05 was taken as the criteria. Different colors represent different KEGG pathways, and the names of KEGG pathways are listed in the figure.

To further analyze the role of SRP-related genes in SSc-PH, we performed unsupervised clustering of the PBMC samples from SSc-PH patients in the training set using the expression values of 30 DE-SRPGs with the R package, “ConsensusClusterPlus.” The consensus matrix indicates that at *k* = 2, the number of patients in each cluster was equally distributed, none of the clusters contained abnormally high or abnormally low numbers of patients, and the correlation between the two clusters was low (Figure 3A). When *k* = 2, the CDF curve was flat (Figure 3B). Figure 3C shows the variations in the area under the CDF curve for *k* = 2–9. Finally, SSc-PH patients from the training set were categorized into two clusters: SRPcluster A and SRPcluster B.

We performed dimensionality reduction of the training set using tSNE and UMAP based on the expression of DE-SRPGs. The tSNE plot (Supplementary Figure S1A) and the UMAP plot (Supplementary Figure S1B) indicated that SRPcluster A was closer to the control group than SRPcluster B, suggesting that SRPcluster A may be an SSc-PH subtype closer to the control group in the two clusters.

### SRP-related pathways and immune infiltration

We performed gene enrichment analysis to explore the potential signaling pathways involved in the SRP gene signature. GSEA indicated that in SRPcluster A, “primary immunodeficiency,” “ribosome,” “RNA degradation,” “spliceosome,” and “T cell receptor signaling pathway” were the significant processes (Figure 3D). In SRPcluster B, the major processes included the “chemokine signaling pathway,” “complement and coagulation cascades,” “Leishmania infection,” “lysosome,” and “regulation of actin cytoskeleton” (Figure 3E).

**FIGURE 4:**
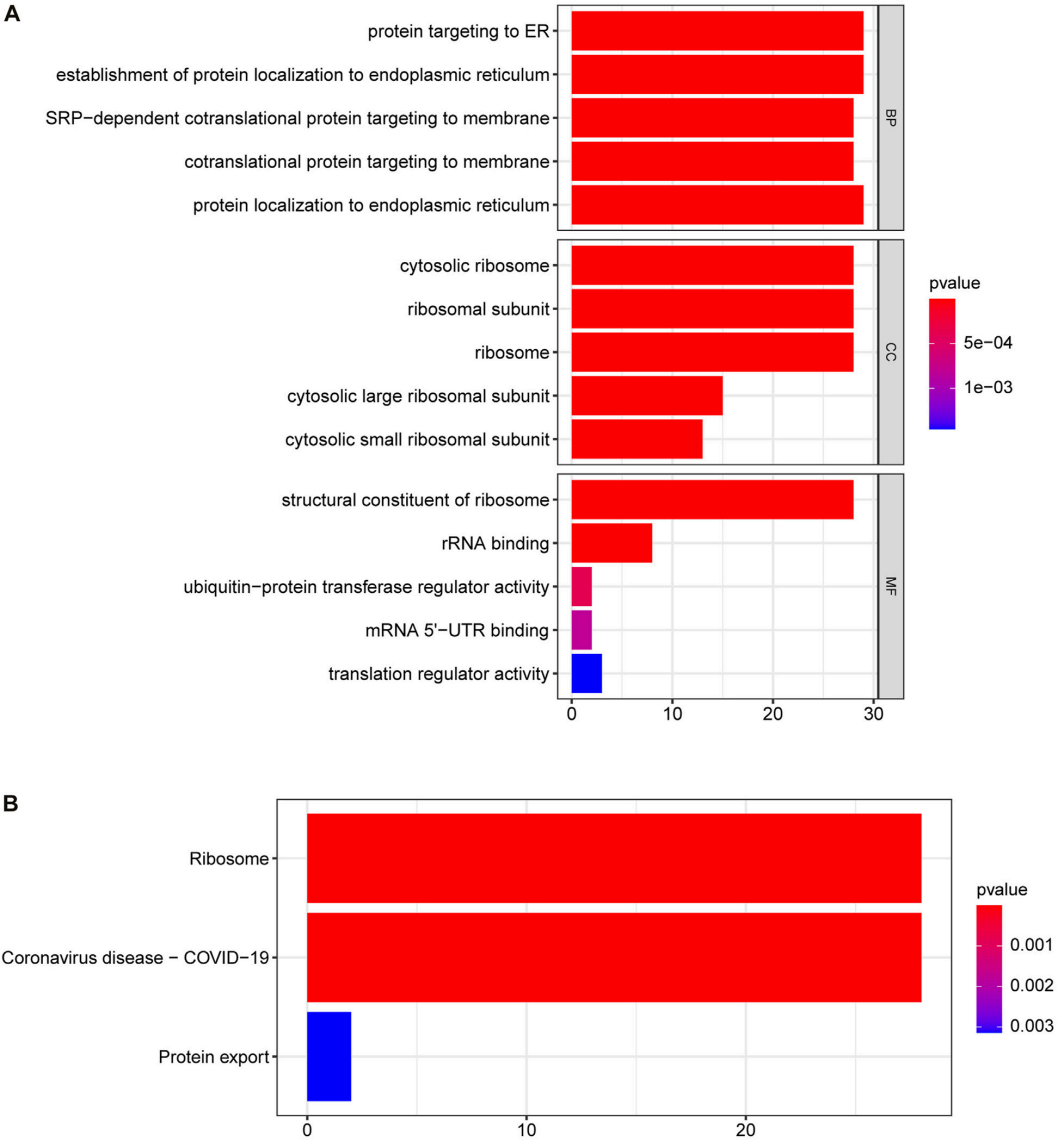
GO annotation and KEGG enrichment analysis of 30 DE-SRPGs. **(A)** Top five enriched biological processes, cellular components, and molecular functions. **(B)** The significantly enriched KEGG pathways. Using a *p*-value <0.05 as criteria. BP, biological process; CC, cellular component; MF, molecular function.

To further explore the functions of the DE-SRPGs, we performed GO annotation (Figure 4A) and KEGG enrichment analysis (Figure 4B) on 30 DE-SRPGs. Supplementary Table S4 shows the complete GO annotation results, and Supplementary Table S5 shows the complete KEGG enrichment analysis results. The GO annotation results suggested that the 30 DE-SRPGs were predominantly enriched in functions and pathways associated with SRP. The most abundant GO biological process (BP) was protein targeting to ER (Figure 4A). Meanwhile, the most abundant GO cellular component (CC) was cytosolic ribosome (Figure 4A), and the most abundant GO molecular function (MF) was structural constituent of ribosome (Figure 4A). The results of the KEGG enrichment analysis indicated that 30 DE-SRPGs were significantly enriched in three signaling pathways: ribosome, coronavirus disease—COVID-19, and protein export (Figure 4B).

**FIGURE 5:**
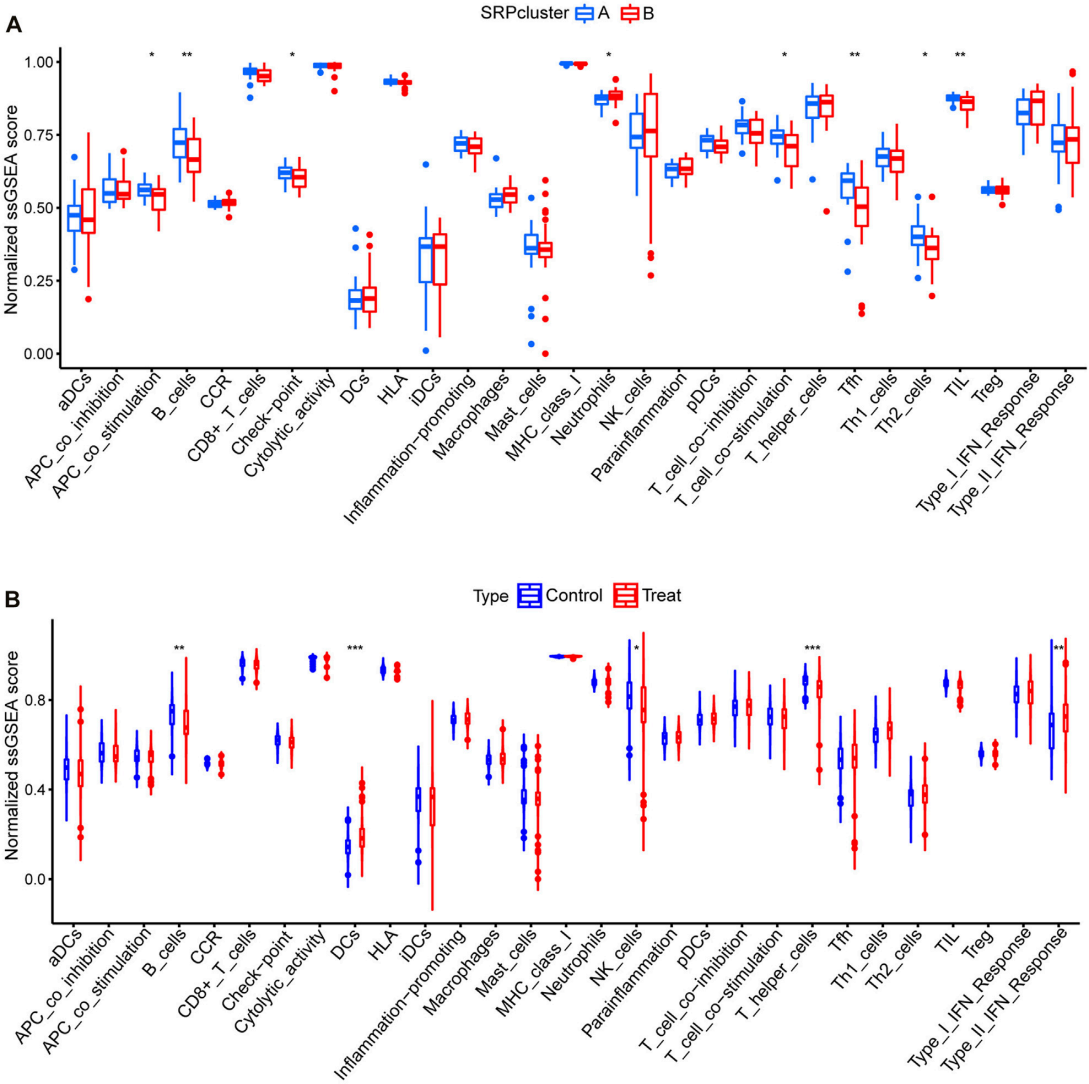
ssGSEA for 29 immune gene sets. **(A)** Comparison of normalized ssGSEA scores of 29 immune gene sets between SRPcluster A and SRPcluster B. **(B)** Comparison of normalized ssGSEA scores of 29 immune gene sets between treatment and control groups. *, *p* < 0.05; **, *p* < 0.01; ***, *p* < 0.001.

**FIGURE 6:**
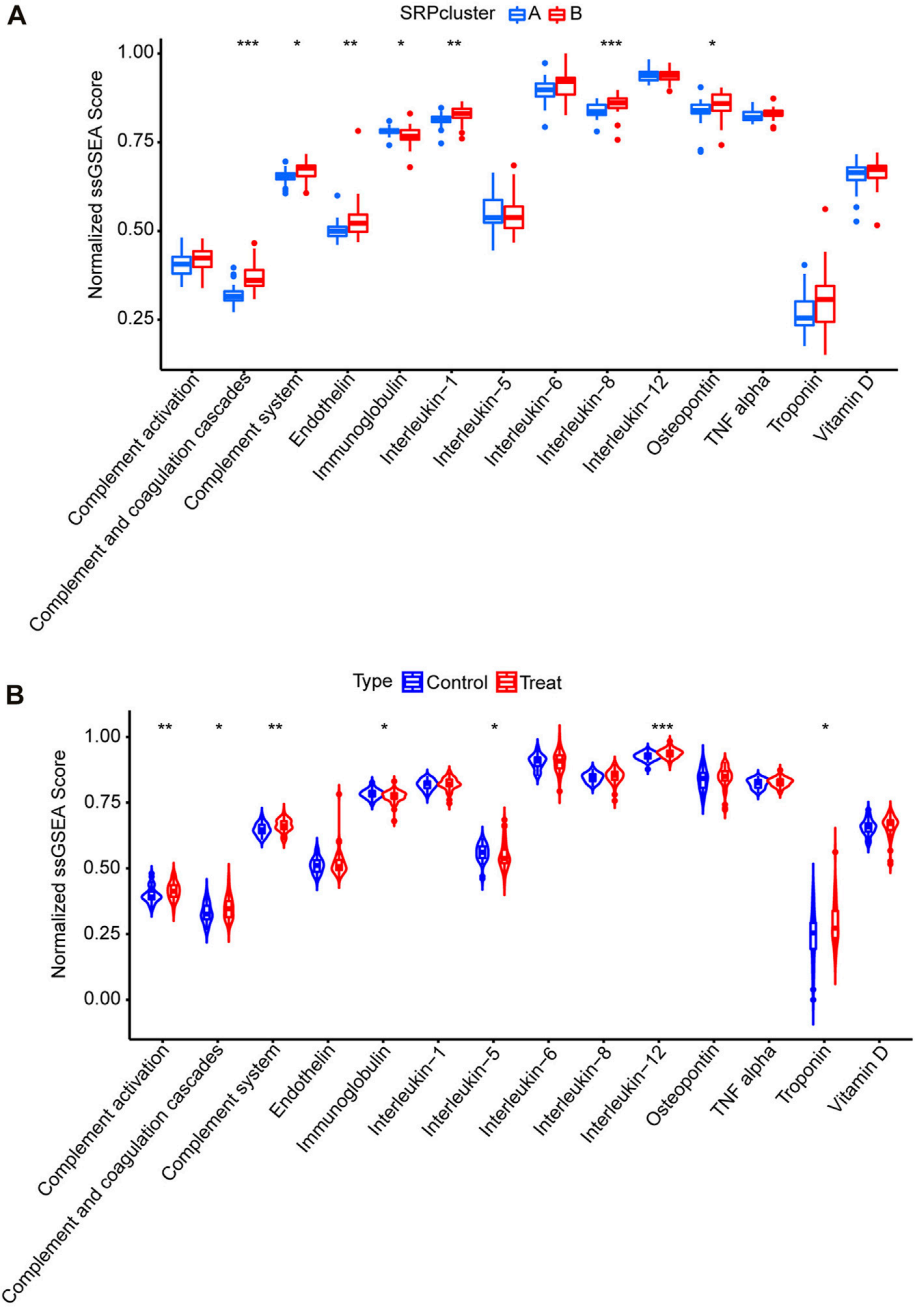
ssGSEA for14 SSc-PH-related pathways. **(A)** Comparison of normalized ssGSEA scores of 14 SSc-PH-related pathways between SRPcluster A and SRPcluster B. **(B)** Comparison of normalized ssGSEA scores of 14 SSc-PH-related pathways between treatment and control groups. *, *p* < 0.05; **, *p* < 0.01; ***, *p* < 0.001.

Subsequently, to determine the relationships among the DE-SRPG expression patterns and immune characteristics, we quantified the normalized ssGSEA scores of typical immune cells and pathways (Figure 5). Between SRPcluster A and SRPcluster B, there were a series of immune cells and pathways that were significantly upregulated in SRPcluster A, including APC costimulation, B cells, T-cell costimulation, T follicular helper cells (Tfh), and tumor-infiltrating lymphocytes (TIL) (Figure 5A). B cells, dendritic cells (DCs), NK cells, T helper cells, and the type II IFN response showed significant differences between the control group (SSc patients without pulmonary hypertension) and treatment group (SSc-PH patients) (Figure 5B). Among the 14 SSc-PH-related pathways, complement and coagulation cascades, complement system, endothelin, interleukin-1, interleukin-8, and osteopontin were significantly upregulated while immunoglobulin was significantly downregulated in SRPcluster B compared with SRPcluster A (Figure 6A). Complement activation, complement and coagulation cascades, complement system, interleukin-12, and troponin were significantly upregulated, while immunoglobulin and interleukin-5 were significantly downregulated in the treatment group compared to the control group (Figure 6B).

### Identification and validation of SRP-related diagnostic genes for SSc-PH based on machine learning

**FIGURE 7:**
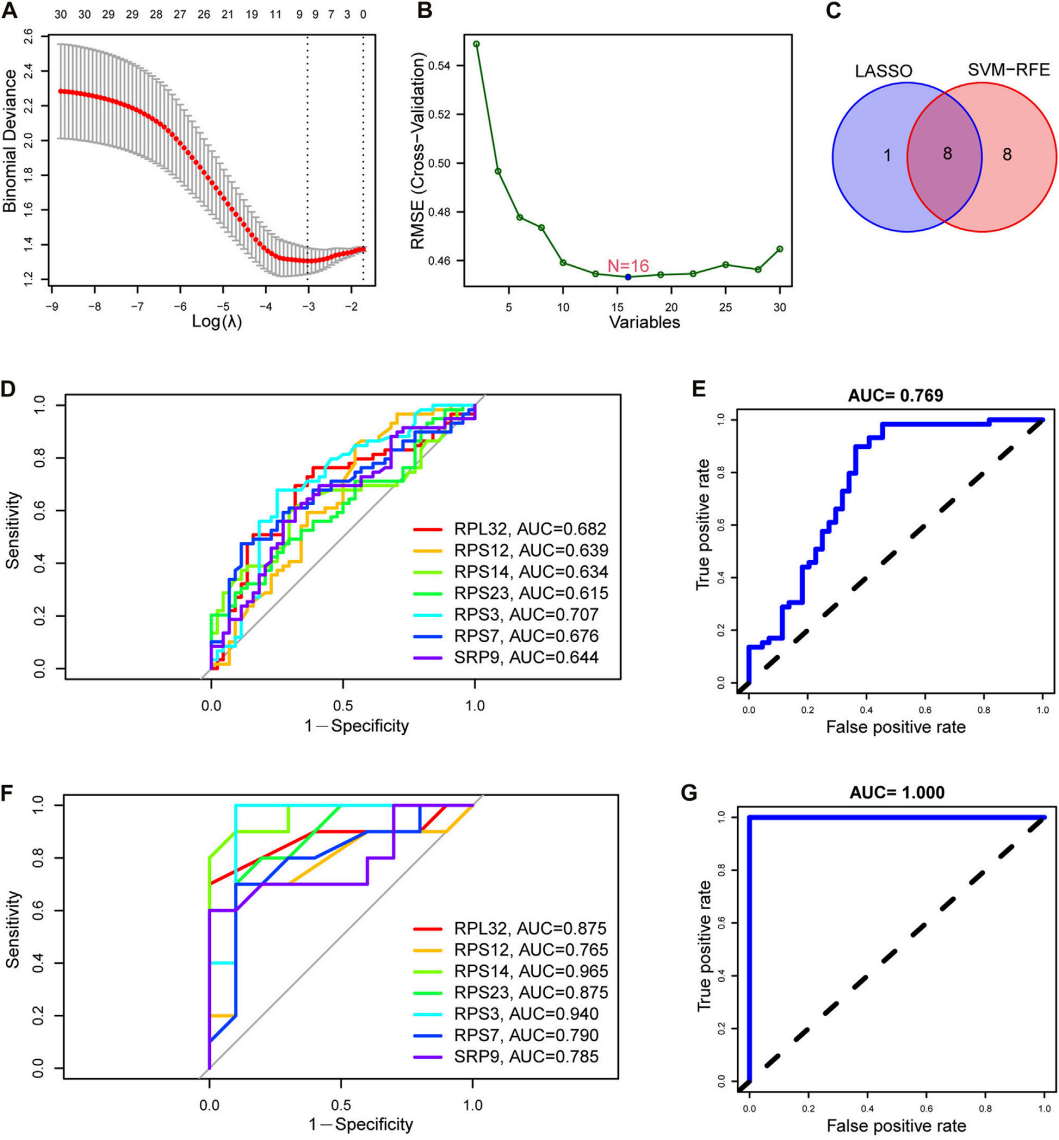
Identification and validation of SRP-DGs. **(A)** Screening of SRP-associated diagnostic markers using the LASSO regression algorithm. **(B)** Screening of SRP-associated diagnostic markers using the SVM-RFE algorithm. **(C)** Venn diagram showing the intersection of SRP-associated diagnostic markers screened by both algorithms. **(D)** ROC curves for the seven SRP-DGs in the training set. **(E)** The ROC curve for the combined diagnosis of seven SRP-DGs in the training set. **(F)** ROC curves for the seven SRP-DGs in the test set. **(G)** The ROC curve for the combined diagnosis of seven SRP-DGs in the test set.

We further screened the SRP-related diagnostic genes (SRP-DGs) for SSc-PH in the DE-SRPGs with two machine learning algorithms. The results showed that we identified nine SRP-related potential diagnostic markers with the LASSO regression algorithm (Figure 7A). Meanwhile, 16 SRP-related potential diagnostic markers were identified by the SVM-RFE algorithm (Figure 7B). Finally, they were intersected to obtain eight SRP-related diagnostic genes (SRP-DGs), namely, RPL10, RPL32, RPS12, RPS14, RPS23, RPS3, RPS7, and SRP9 (Figure 7C).

From the previous results, we learned that the expression of RPL10 was missing in the test set (GSE22356) among the eight SRP-DGs due to platform differences. This leads to the fact that if the diagnostic model is constructed using all eight SRP-DGs, it will cause inconsistencies between the model in the training set and test set and cause difficulties in validation. Therefore, we used seven SRP-DGs, namely, RPL32, RPS12, RPS14, RPS23, RPS3, RPS7, and SRP9, to construct the diagnostic model for SSc-PH.

Subsequently, we plotted the ROC curves for the seven SRP-DGs. The results showed that RPL32, RPS12, RPS14, RPS23, RPS3, RPS7, and SRP9 had good diagnostic efficacy in the training set, with area under the ROC curve (AUC) values of 0.682, 0.639, 0.634, 0.615, 0.707, 0.676, and 0.644, respectively (Figure 7D). When the seven SRP-DGs were combined into one signature, the AUC value was 0.769 (Figure 7E). We also validated the diagnostic efficacy of the seven SRP-DGs in the test set. The results showed that the AUC values of RPL32, RPS12, RPS14, RPS23, RPS3, RPS7, and SRP9 were 0.875, 0.765, 0.965, 0.875, 0.940, 0.790, and 0.785 in the test set, respectively (Figure 7F). When the seven SRP-DGs were combined into one signature, the AUC value was 1.000 (Figure 7G).

As a result, these seven SRP-DGs can effectively distinguish SSc-PH patients from SSc patients without pulmonary hypertension and have better diagnostic efficacy when combined.

In addition, we compared the expression levels of seven SRP-DGs in SRPcluster A, SRPcluster B, and the control group (Supplementary Figure S2A–G). The results indicated that the expression levels of seven SRP-DGs in SRPcluster A were closer to those in the control group than in SRPcluster B.

### Generation and analysis of the SRP scoring system

**FIGURE 8:**
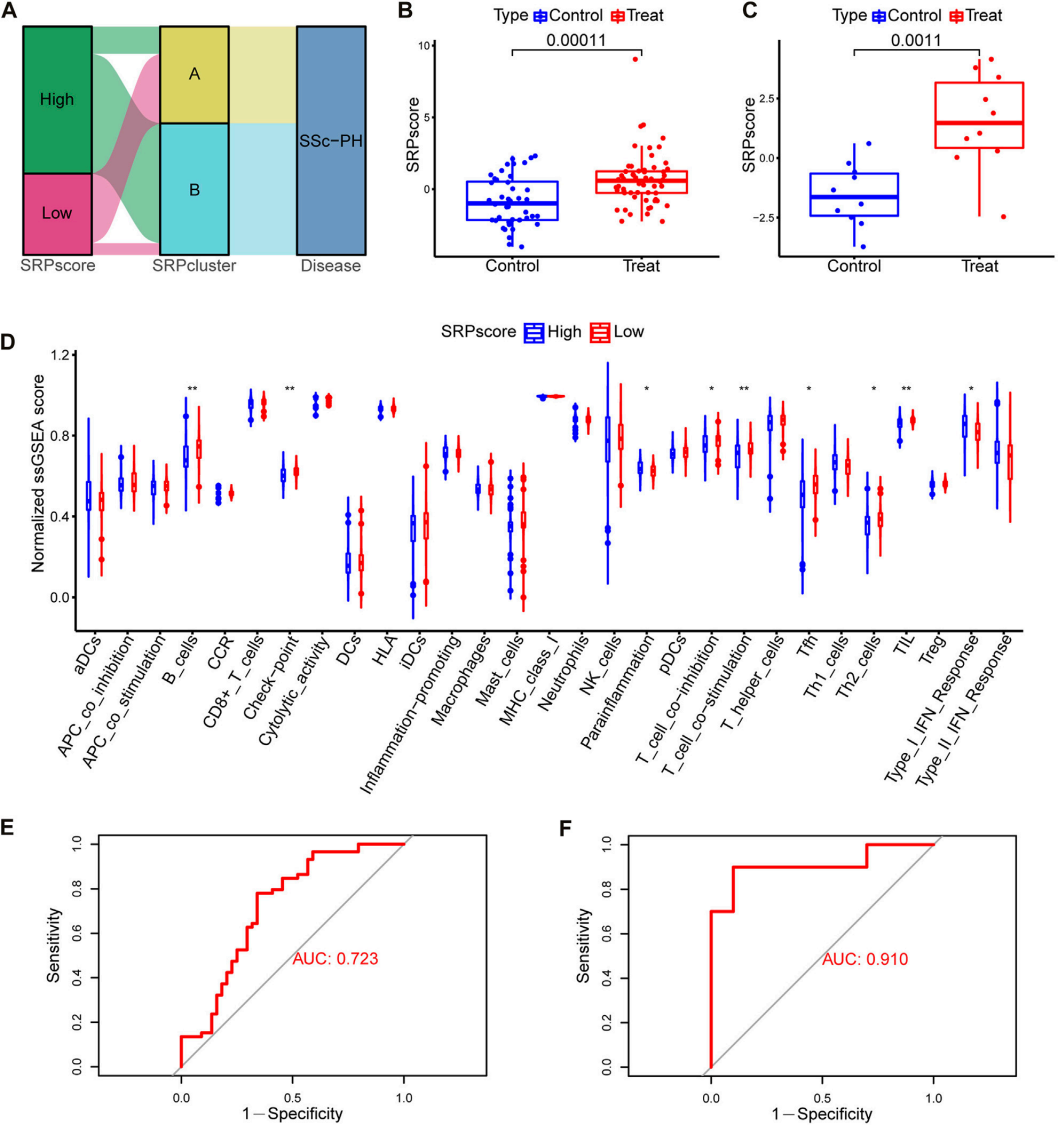
Construction and analysis of the SRPscore. **(A)** An alluvial plot showing SRPcluster, SRPscore, and disease changes. **(B)** SRPscore difference between the treatment and control groups in the training set. **(C)** SRPscore difference between the treatment and control groups in the test set. **(D)** Differences in normalized ssGSEA scores for the 29 immune gene sets between different SRPscore groups. **(E)** The ROC curve of the SRPscore in the training set. **(F)** The ROC curve of the SRPscore in the test set. *, *p* < 0.05; **, *p* < 0.01; ***, *p* < 0.001.

To more accurately quantify the personalized SRP-related gene expression pattern of each patient, we constructed a scoring system, SRPscore, based on the seven SRP-DGs. Supplementary Table S6 shows the SRPscore values of the samples in the training set, and Supplementary Table S7 shows the SRPscore values of the samples in the test set. We visualized the attributes of each SSc-PH patient using alluvial plots (Figure 8A). The results showed that most SSc-PH patients with high SRPscore values belonged to SRPcluster B, whereas the majority of SSc-PH patients with low SRPscore values belonged to SRPcluster A (Figure 8A). The SRPscore values in the treatment group were significantly higher than those in the control group in both the training and test sets (Figures 8B,C).

Subsequently, we investigated whether patients in the high SRPscore group had a different type of immune cell infiltration than those in the low SRPscore group. The ssGSEA results showed that the patients in the high SRPscore group had significantly lower normalized ssGSEA scores for “B cells,” “check-point,” “T cell co-inhibition,” “T cell co-stimulation,” “Tfh” (T follicular helper cells), “Th2” (T helper 2 cells) and “TIL” (tumor-infiltrating lymphocytes) but significantly higher normalized ssGSEA scores for “parainflammation” and “type I IFN response” than those in the low SRPscore group (Figure 8D).

Then, we tested whether the SRPscore values could be used as an independent diagnostic biomarker to distinguish SSc-PH patients from SSc patients without pulmonary hypertension. The results showed that the AUC values of the SRPscore were 0.723 and 0.910 in the training set (Figure 8E) and test set (Figure 8F), respectively, thus validating the diagnostic efficacy of the SRPscore.

We compared the SRPscore values in SRPcluster A, SRPcluster B, and the control group (Supplementary Figure S2H). The results showed that the SRPscore values in SRPcluster A were closer to the control group than SRPcluster B, further demonstrating that the SRP-related gene expression patterns in SRPcluster A were closer to that of the control group.

### Construction of the SSc-PH nomogram model

**FIGURE 9:**
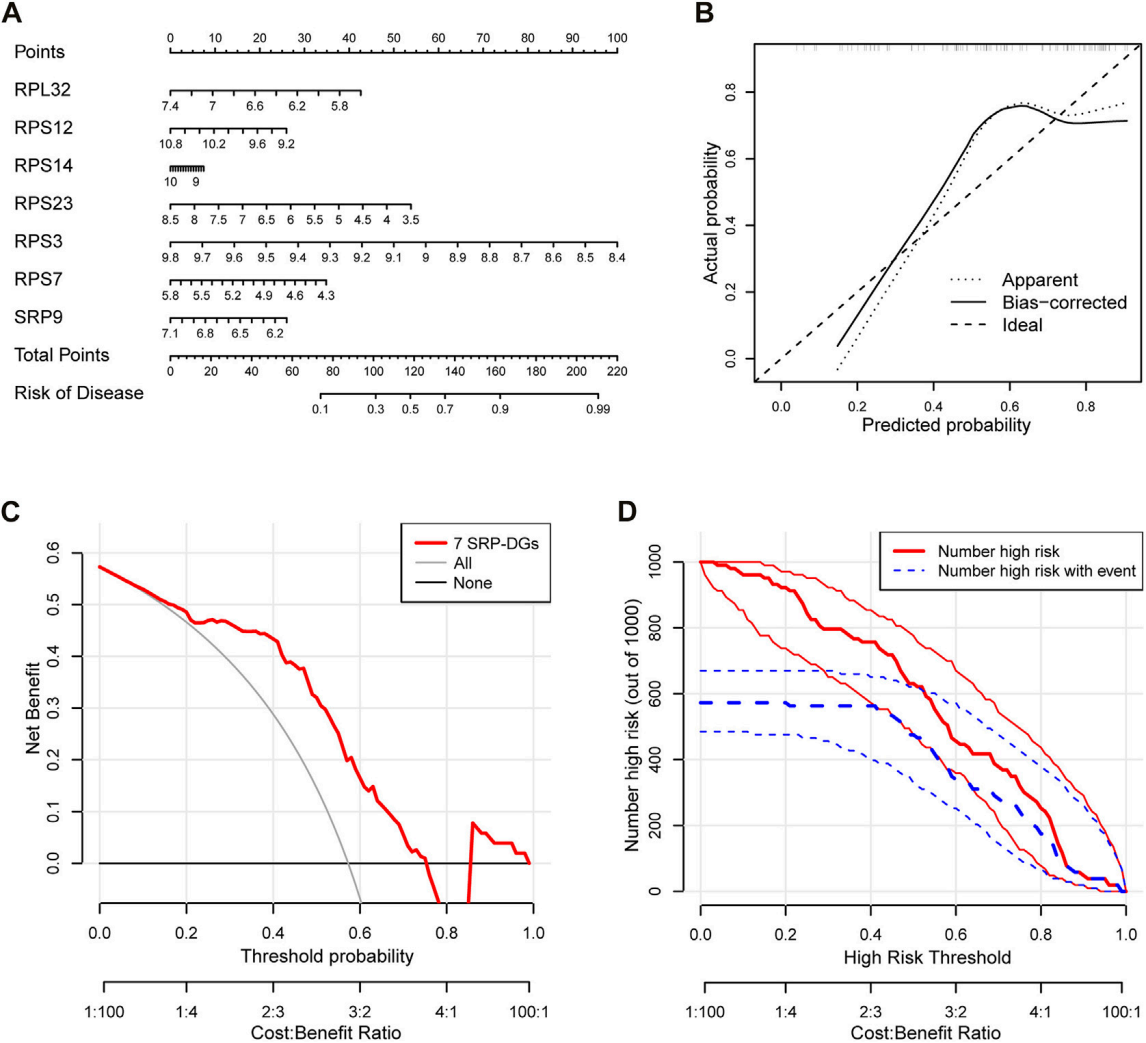
Construction of an SSc-PH diagnostic model based on seven SRP-DGs. **(A)** Nomogram for predicting SSc-PH risk based on seven SRP-DGs. **(B)** The calibration curve showing the accuracy of predicting SSc-PH. **(C)** The decision curve for analyzing the benefits of the diagnostic model. **(D)** The clinical impact curve showing the predicted probability of the diagnostic model.

To further investigate the relationships among the SRP-DGs and risk of SSc-PH, we constructed a nomogram model using seven SRP-DGs (RPL32, RPS12, RPS14, RPS23, RPS3, RPS7, and SRP9) to predict the risk of pulmonary hypertension complications in patients with SSc (Figure 9A). The calibration curve indicated that the nomogram model was relatively accurate in predicting SSc-PH (Figure 9B). The decision curve demonstrated that the predictions made using the nomogram model could be beneficial to patients (Figure 9C). Moreover, the clinical impact curve indicated the good predictive capacity of the nomogram model (Figure 9D).

### Prediction performances of the ANN model in the training and test sets

**FIGURE 10:**
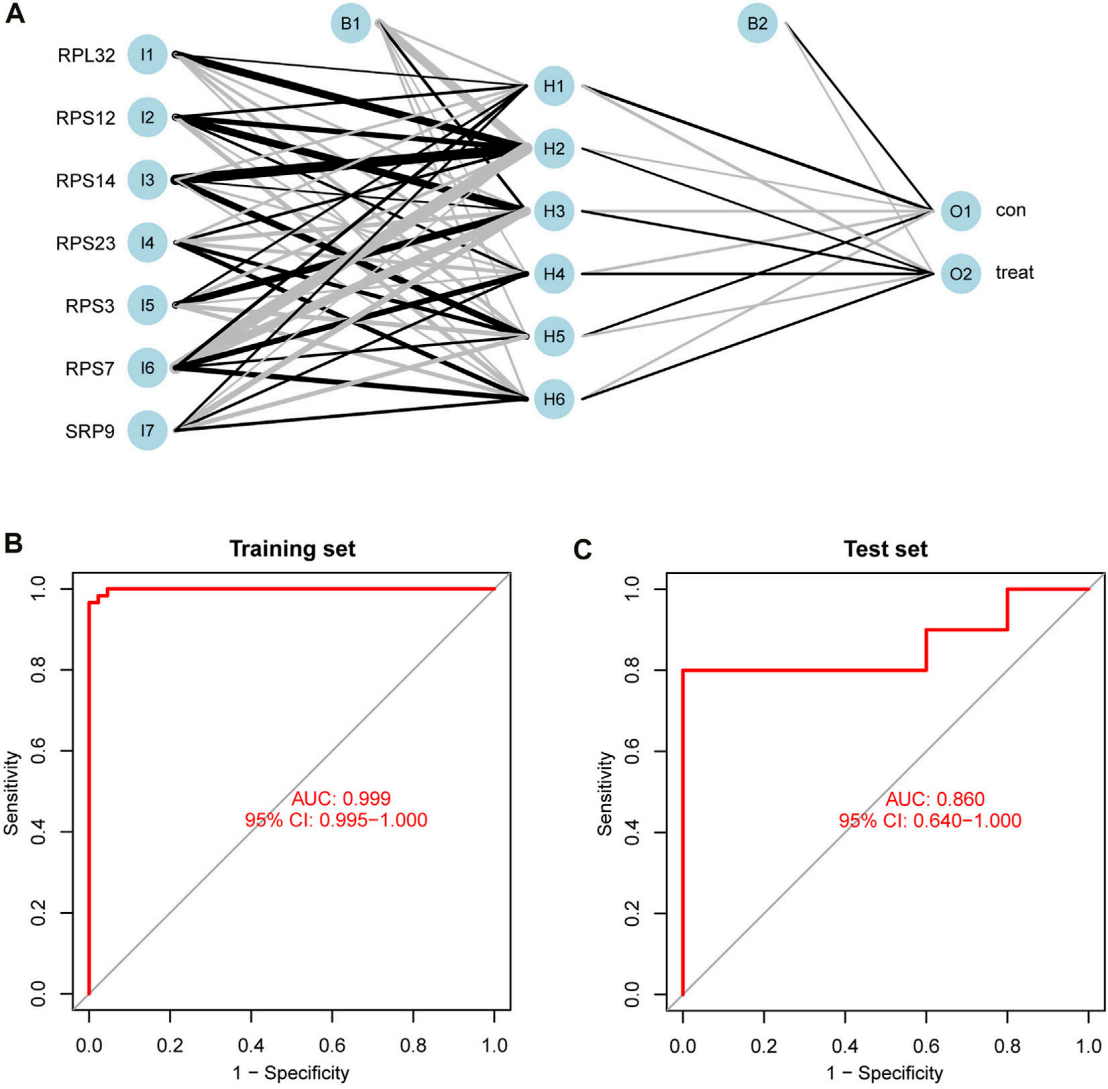
Construction and validation of ANN. **(A)** The process of constructing ANN. **(B)** ROC curve of ANN in the training set with an AUC value of 0.999. **(C)** ROC curve of ANN in the test set with an AUC value of 0.860. 95% CI: 95% confidence interval.

After normalizing the expressions of the seven SRP-DGs using the min-max method, we constructed an ANN model to predict whether the samples belonged to the control group or treatment group (Figure 10A). The output results of the artificial neural network are shown in Supplementary Table S8. Then, we compared the prediction results of the ANN model with the actual grouping information and evaluated the model prediction accuracy. Subsequently, we performed ROC to evaluate the prediction performances of the ANN in the training and test sets. The results showed that the AUC values for the training and test sets were 0.999 and 0.860, respectively (Figures 10B,C). Table 2 shows the complete results of the prediction accuracies and AUC values of the ANN for the training and test sets. Overall, the ANN model was credible and has potential as an independent diagnostic predictor of SSc-PH. The results also confirmed that SRP-related genes are likely to play an essential role in the pathogenesis of SSc-PH.

**TABLE 2:** Neural network diagnostics for the training and test sets.

		Training set		Test set	
		Control	Treat	Control	Treat
Prediction results	Control	43	1	5	2
	Treat	1	58	5	8
	Control accuracy	0.977		0.500	
	Treat accuracy	0.983		0.800	
	AUC	0.999		0.860	

### Correlation analysis of seven SRP-DGs with immune characteristics and SSc-PH-related pathways

**FIGURE 11:**
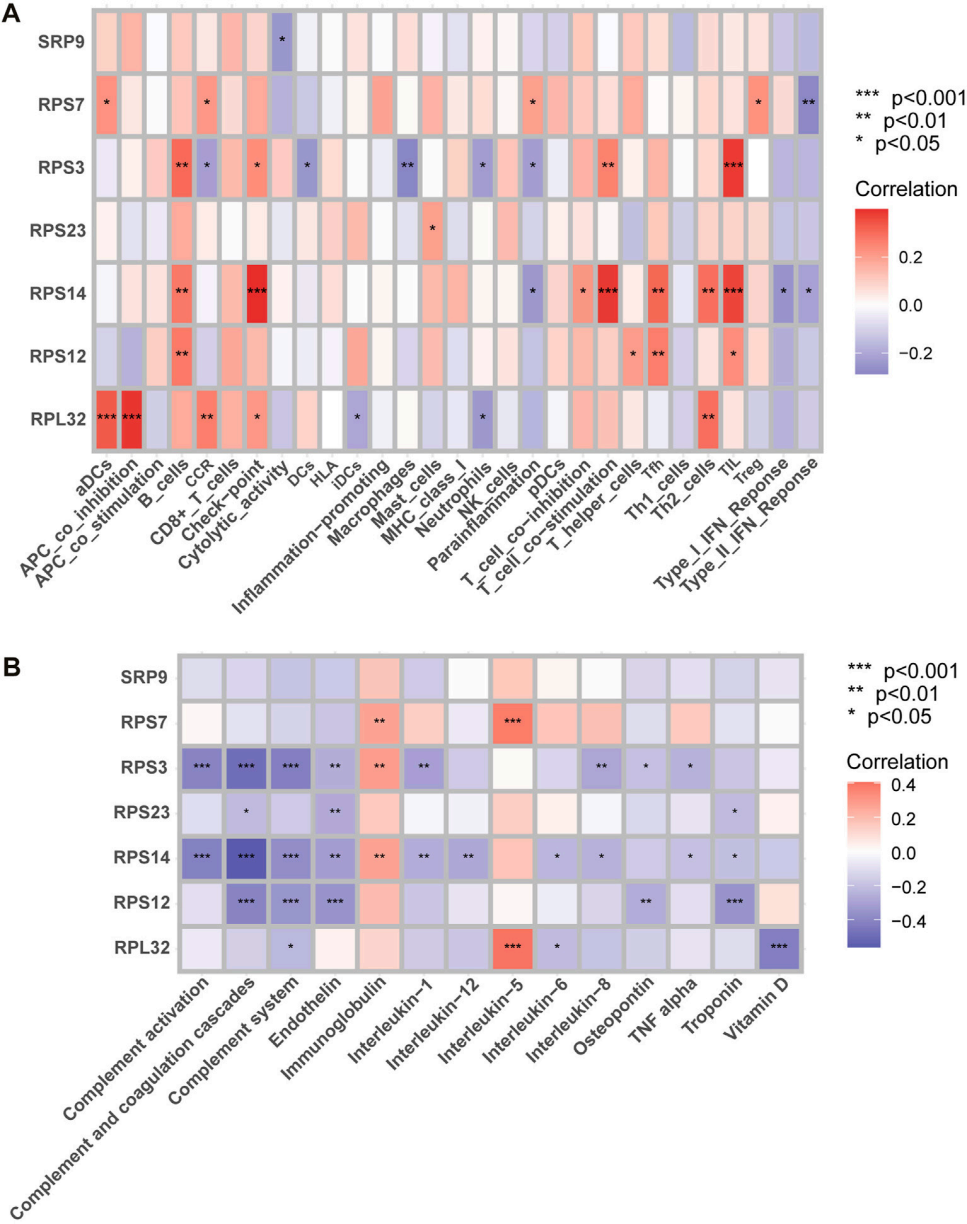
Correlation analysis. **(A)** Correlation analysis between SRP-DGs and immune characteristics. **(B)** Correlation analysis between SRP-DGs and SSc-PH-related pathways. Red represents positive correlations, and purple represents negative correlations. The deeper the color, the greater the correlation. *, *p* < 0.05; **, *p* < 0.01; ***, *p* < 0.001.

We calculated Spearman correlation coefficients and *p* values for the expressions of seven SRP-DGs with normalized ssGSEA scores for 29 immune gene sets and 14 SSc-PH-related pathways. The results showed that the seven SRP-DGs were related to a series of immune cells, functions, and SSc-PH-related pathways (Figure 11).

For example, RPL32 was significantly positively correlated with “aDCs” (activated dendritic cells), “APC co-inhibition” (Figure 11A), and “interleukin-5” (Figure 11B) while significantly negatively correlated with “vitamin D” (Figure 11B) (*p* < 0.001). RPS14 was significantly positively correlated with “check-point,” “T cell co-stimulation,” and “TIL” (Figure 11A) while significantly negatively correlated with “complement activation,” “complement and coagulation cascades,” and “complement system” (Figure 11B) (*p* < 0.001). RPS3 was significantly positively correlated with “TIL” (Figure 11A) while significantly negatively correlated with “complement activation,” “complement and coagulation cascades,” and “complement system” (Figure 11B) (*p* < 0.001). RPS12 was significantly negatively correlated with “complement and coagulation cascades,” “complement system,” “endothelin,” and “troponin” (Figure 11B) (*p* < 0.001). RPS7 was significantly positively correlated with “interleukin-5” (Figure 11B) (*p* < 0.001). The results suggest that SRP-related genes may influence the immune microenvironments of SSc and SSc-PH patients and disease progression by regulating dendritic cells, T cells, and B cells. Meanwhile, SRP-related genes may regulate functions and pathways that play essential roles in pulmonary vascular remodeling, such as complement activation, the complement system, complement and coagulation cascades, endothelin, troponin, interleukin, and vitamin D.

### Further exploration of the functions of the seven SRP-DGs

Supplementary Figure S3 shows the results of single gene batch correlation analysis-based GSEA for RPL32, RPS3, RPS7, and RPS12. Supplementary Figure S4 shows the results of single gene batch correlation analysis-based GSEA for RPS14, RPS23, and SRP9.

The results indicated that all seven SRP-DGs might inhibit heme metabolism. RPS3, RPS7, RPS12, RPS14, RPS23, and SRP9 might inhibit coagulation. RPL32, RPS3, RPS12, RPS14, RPS23, and SRP9 might be involved in the inhibition of interferon-alpha response. RPS3, RPS12, RPS14, RPS23, and SRP9 are likely to be involved in suppressing the interferon-gamma response. RPL32, RPS3, RPS12, and RPS14 may be involved in inhibiting the complement system. RPS3, RPS7, RPS12, and RPS14 potentially activate DNA repair. In addition, the results demonstrated that SRP-DGs might be linked to a series of functions and pathways such as epithelial-mesenchymal transition, IL-6/JAK/STAT3 signaling, TNF alpha signaling, mTORC1, oxidative phosphorylation, inflammatory response, and apoptosis.

### Screening for drugs targeting SRP-DGs.

Based on the DSigDB database, we used the Enrichr platform to identify drug molecules associated with the seven SRP-DGs with a *p*-value < 0.05. The combined scores reflect the correlations between drugs and genes, and higher combined scores indicate stronger correlations between drugs and genes. Table 3 lists the drugs with the top ten rankings in their combined score and *p* values < 0.05. The results indicate that 2,6-DICHLORO-4-NITROPHENOL CTD 00000815 has a strong affinity for RPS3, while Fenbuconazole CTD 00004512 is likely to have a regulatory effect on RPS7.

**TABLE 3:** Potential drugs that may have regulatory effects on the seven SRP-DGs.

Drug	*P*-value	Combined score	Target genes
2,6-DICHLORO-4-NITROPHENOL CTD 00000815	0.0076757	771.8855079	RPS3
Fenbuconazole CTD 00004512	0.0125339	416.2019442	RPS7
Artesunate CTD 00001840	0.0128802	402.1028593	RPS12
Beryllium sulfate CTD 00001005	0.0135723	376.3201193	RPS7
okadaic acid CTD 00007275	0.0344842	112.7746184	RPS12
3,3′-Diindolylmethane CTD 00000841	0.0361814	105.7945348	RPS3
ursodiol CTD 00006973	0.0476541	72.9883936	RPL32
Disodium selenite CTD 00007229	0.0091245	47.9593338	RPL32, RPS7, RPS3
hydralazine CTD 00006108	0.0277972	35.5378836	SRP9, RPS12
estradiol CTD 00005920	0.0439133	15.0614469	RPS14, RPL32, RPS7, RPS3

## Discussion

The DETECT and ASIG algorithms are routine methods for screening SSc-PH, but the heterogeneity of patient clinical outcomes may limit their application. The entry criteria for the DETECT study were DLCO <60% and SSc durations longer than 3 years, which were designed to ensure that high-risk patients were included; however, in clinical practice, this may have resulted in patients with DLCO ≥60% and patients with early SSc being missed (Hao et al., 2015; Young et al., 2021). It has been shown that the ASIG algorithm has higher specificity than the DETECT algorithm, but it is likely to miss WHO Group 2 PH patients (Hao et al., 2015). Its applicability in different racial populations remains to be explored (Coirier et al., 2021). Therefore, finding new genetic biomarkers and developing more straightforward and objective diagnostic models are necessary. Meanwhile, there is increasing evidence that SRP depletion plays an integral role in autoimmune diseases, cancer, and neurodegenerative diseases (Kellogg et al., 2022). In this study, we identified complex correlations between SRP-related genes and SSc-PH diagnosis. We developed a diagnostic model for SSc-PH containing seven SRP-related genes by using LASSO regression, SVM-RFE, and ANN to effectively distinguish SSc-PH patients from SSc patients and guide SSc-PH diagnosis and treatment.

We obtained 30 DE-SRPGs. In the training set, all 30 DE-SRPGs were significantly downregulated in SSc-PH. Meanwhile, in the test set, except for 6 DE-SRPGs that were missing due to platform differences, all other 24 DE-SRPGs were also significantly downregulated in SSc-PH. This suggests that SRP-dependent cotranslational protein targeting may be dysfunctional in SSc-PH. At the molecular cell biology level, the characteristics of PH include endoplasmic reticulum stress, mitochondrial dysfunction, DNA damage, and transcription factor dysregulation (Lopez-Crisosto et al., 2021). During endoplasmic reticulum stress, the XBP1 protein has a role in increasing the size of the endoplasmic reticulum and reducing endoplasmic reticulum stress. However, the XBP1 protein can only be synthesized when a portion of XBP1 mRNA is cleaved (Park et al., 2021). To cleave this portion of XBP1 mRNA, Ire1α first localizes to the Sec61 channel on the endoplasmic reticulum membrane, while the XBP1 protein is cotranslationally targeted to the Sec61 channel by SRP, and this portion of XBP1 mRNA is cleaved by Ire1α (Plumb et al., 2015). SRP depletion, SRP receptor depletion, and Sec61 depletion all block the above processes. Furthermore, upon SRP depletion, proteins that should be cotranslationally targeted to the endoplasmic reticulum may be mislocalized to the mitochondria, directly leading to mitochondrial dysfunction (Costa et al., 2018). This may also be one reason why SRP depletion leads to SSc-PH. The relationship of SRP with DNA damage and transcription factor dysregulation remains to be explored.

To our surprise, the “coronavirus disease—COVID-19,” was identified in the KEGG enrichment analysis results. It has been demonstrated that two severe acute respiratory syndrome coronavirus 2 (SARS-CoV-2) viral proteins, NSP8 and NSP9, can bind to the 7SL RNA component of SRP, disrupting the function of SRP and inhibiting the transport of membrane proteins, thereby suppressing host immune defenses (Banerjee et al., 2020). Whether this process can lead to pulmonary hypertension in patients with coronavirus disease 2019 (COVID-19) and the potential common pathogenic mechanisms of SSc-PH and COVID-19 remain to be investigated.

In terms of phenotyping, based on different clinical features and pathogenesis, the World Health Organization (WHO) classified PH into five groups (Simonneau et al., 2019). Each group requires a different treatment protocol. SSc-PH may be caused by primary vasculopathy of the small pulmonary arteries (Group 1), left heart failure (Group 2), or interstitial lung disease (Group 3) (Attanasio et al., 2020). However, due to the complexity of SSc-PH, multiple groups of PH are likely to overlap in a single SSc-PH patient, making it challenging to develop a treatment protocol (Attanasio et al., 2020). In this study, we clustered SSc-PH patients based on the SRP-related genes and developed SRPscore, an SRP-related scoring system, to explore the differences in signaling and immune infiltration in SSc-PH patients with different clusters and different scores, which can provide a basis for precision and personalized medicine for SSc-PH. By performing dimensionality reduction by tSNE and UMAP and comparing the expression of SRP-DGs and SRPscore values in SRPcluster A, SRPcluster B, and the control group, we found that the expression patterns of SRP-related genes in SRPcluster A were closer to those in the control group compared with SRPcluster B. Meanwhile, the ssGSEA results indicated that between SRPcluster A and SRPcluster B, the immune responses might be more active in SRPcluster A, while the pathways related to SSc-PH were likely to be more activated in SRPcluster B. However, whether the expression patterns of SRP-related genes and SRPscore are associated with the progression of SSc-PH and have the potential to predict the prognosis of SSc-PH patients needs to be further investigated, which is the direction of a future study.

We identified seven SRP-DGs and constructed a nomogram and ANN model for SSc-PH predictions based on our findings. Among the seven SRP-DGs, downregulation of SRP9 is related to the development and progression of multiple types of cancer. It has been indicated that in breast cancer, deficiencies of SRP9 and SRP14 activate RIG-1, which further causes an interferon response, increases inflammation, and leads to breast cancer metastasis (Nabet et al., 2017). In addition, SRP9 has shown potential as a prognostic marker for colorectal cancer and non-Hodgkin’s lymphoma (Lee et al., 2017; Matsumoto et al., 2021). Among the seven SRP-DGs, RPL32, RPS12, RPS14, RPS23, RPS3, and RPS7 all encode ribosomal proteins. Among them, the protein encoded by RPL32 is part of the large (60S) subunit of ribosomes, while the proteins encoded by RPS12, RPS14, RPS23, RPS3, and RPS7 are involved in structuring the small (40S) subunit of ribosomes (Kang et al., 2021). Ribosomal proteins may regulate SRP-mediated cotranslational protein targeting in two ways. On the one hand, the S domain of SRP binds to the 60S subunit of the ribosome, during which some ribosomal proteins inside the ribosomal tunnel reach the outside of the ribosome, affecting the interaction of the ribosome-nascent chain complex with cytosolic targeting factors, thus regulating SRP and influencing Sec61 channel opening and closing (Schäuble et al., 2012; Denks et al., 2017; Pool, 2022). On the other hand, the N domain of SRP54 also contacts ribosomal proteins, facilitating more timely and efficient recognition of signals, while blocking this process would lead to deficiencies in SRP-dependent cotranslational protein targeting (Dalley et al., 2008). RPS14 haploinsufficiency is associated with myelodysplastic syndrome with chromosome 5q deletion (Schneider et al., 2016). RPS7 may inhibit glycolysis through HIF-1α-related signaling and thus play a protective role in colorectal cancer (Zhang et al., 2016).

Studies have shown that SRP proteins that undergo immune system attack can cause lung and heart diseases (Kassardjian et al., 2015; Milone, 2017). Meanwhile, in a cohort of 460 patients, researchers observed that patients with anti-SRP antibodies developed lung diseases more frequently than those with anti-HMGCR antibodies (Watanabe et al., 2016). Case reports by Below and Bashir (2021) and Baah et al. (2021) also indicated that the early onset of pulmonary hypertension in patients might be associated with SRP proteins. Nevertheless, most of these studies focused on inflammatory myopathies, and the relationships between SRP-DGs and other diseases remain to be explored. Our study suggests that these seven SRP-DGs are important potential biomarkers for SSc-PH, but more studies are needed to validate our results.

Furthermore, we studied the relationships between SRP-DGs and immune characteristics. The results showed that SRP-DGs might affect the immune infiltration microenvironment of SSc-PH by influencing multiple immune cells and pathways, such as activated dendritic cells, B cells, APC coinhibition, and T-cell costimulation. There are few studies on the relationship between SRP and the immune system. It has been demonstrated that anti-SRP antibodies may be involved in the complement cascade and that destruction of SRP subunits by CD5^+^ B cells and CD4^+^ T cells contributes to inflammation (Allenbach et al., 2018; Bergua et al., 2019; Kellogg et al., 2022). Correlation analysis for SRP-DGs and 14 SSc-PH-related pathways revealed that SRP-DGs might involve in complement-related biological processes such as complement and coagulation cascades, complement activation, and the complement system, as well as in the regulation of endothelin, troponin, vitamin D, and interleukins. The crucial role of complement activation in pulmonary hypertension has been clarified. Activation of classical and alternative complement pathways has been reported in perivascular lesions (Frid et al., 2020). Meanwhile, the upregulation of granulocyte-macrophage colony-stimulating factor and proliferation of pulmonary vascular tissue can be found downstream of complement activation (Hu et al., 2020). Endothelin levels can reflect the severity of PH and have the potential to predict the response of SSc-PH patients to bosentan treatment (Kawashiri et al., 2014). Troponin is closely correlated with PH and has been identified as a predictive biomarker of mortality in patients with PH (Odler et al., 2018). Reduced serum vitamin D levels are associated with pulmonary involvement in systemic sclerosis (Groseanu et al., 2016). In addition, studies have shown that plasma interleukin-1β, interleukin-6, and interleukin-8 levels are significantly increased in SSc-PH patients, but interleukin-5 levels are not statistically different between SSc-PH and SSc patients (Christmann et al., 2011; McMahan et al., 2015).

Single gene batch correlation analysis-based GSEA revealed that SRP-DGs might be mainly involved in heme metabolism, coagulation, interferon-alpha response, interferon-gamma response, complement system, and DNA repair. In the previous paragraph, we discussed the role of complement in the pathogenesis of SSc-PH. Increased heme metabolism might affect mitochondrial respiration and has been reported to be observed in the lung tissue of patients with advanced PH (Sommer et al., 2022). Coagulation processes have been demonstrated to play an essential role in the pathogenesis of PH (Bazan and Fares, 2018). Hyperactivation of coagulation processes and thrombocytopenia can be observed in patients with PH (Vrigkou et al., 2020). However, the use of anticoagulation reduces mortality in idiopathic PH patients but may increase mortality in SSc-PH patients, and the reasons behind this phenomenon need to be investigated (Khan et al., 2018). George et al. (2014) found elevated levels of interferon-alpha and interferon-gamma in SSc-PH patients compared to SSc patients and demonstrated that type I interferon mediates PH through IFNAR1. DNA damage, genomic instability, and dysregulation of the DNA damage response pathway play a crucial role in the pathogenesis of PH (Sharma and Aldred, 2020). Our study reveals that the expression levels of RPS3, RPS7, RPS12, and RPS14 are positively correlated with DNA repair. Nevertheless, whether SRP-related genes can promote DNA repair and the specific mechanisms involved need to be investigated.

Using the Enrichr platform, we conclude that 2,6-dichloro-4-nitrophenol has a strong affinity for RPS3. 2,6-dichloro-4-nitrophenol is a broad-spectrum inhibitor of sulfotransferases. In hepatocytes, pretreatment with 2,6-dichloro-4-nitrophenol may reduce the hepatotoxicity associated with the application of labetalol hydrochloride (Yang L. et al., 2021). However, whether 2,6-dichloro-4-nitrophenol can be used to treat SSc-PH and its possible interaction pattern with RPS3 still need to be corroborated by more studies.

This study has several limitations. With respect to internal validity, regulation of the immune system by SRP and the role of SRP in the pathogenesis of SSc-PH need more research to be substantiated. We did not filter DE-SRPGs by fold change, which may lead to insufficient stability and interpretability of the results. With respect to external validity, the accuracy of the ANN model needs further investigation, and more basic and clinical studies should be conducted to find more straightforward and cost-effective screening methods for SSc-PH.

## Data Availability

The original contributions presented in the study are included in the article/Supplementary Material, further inquiries can be directed to the corresponding author.
