# Supplementary material for: A signal recognition particle-related joint model of LASSO regression, SVM-RFE and artificial neural network for the diagnosis of systemic sclerosis-associated pulmonary hypertension
Source: Front Genet. 2022 Nov 28;13:1078200. doi: 10.3389/fgene.2022.1078200 (PMC9742487; doi:10.3389/fgene.2022.1078200)
Supplement: Supplementary file 1 [file Table8.DOCX]

**Supplementary Table S8. The output results of the artificial neural network.**

| error | 3.799616 |
| --- | --- |
| reached.threshold | 0.009424185 |
| steps | 59747 |
| Intercept.to.1layhid1 | -2.691932 |
| RPL32.to.1layhid1 | 0.09350614 |
| RPS12.to.1layhid1 | 3.414178 |
| RPS14.to.1layhid1 | -2.973215 |
| RPS23.to.1layhid1 | -3.18754 |
| RPS3.to.1layhid1 | 1.886107 |
| RPS7.to.1layhid1 | 5.146176 |
| SRP9.to.1layhid1 | 2.459232 |
| Intercept.to.1layhid2 | -20.21002 |
| RPL32.to.1layhid2 | 17.69987 |
| RPS12.to.1layhid2 | 10.77879 |
| RPS14.to.1layhid2 | 25.31489 |
| RPS23.to.1layhid2 | 3.533146 |
| RPS3.to.1layhid2 | -4.591183 |
| RPS7.to.1layhid2 | -31.37128 |
| SRP9.to.1layhid2 | -0.3991072 |
| Intercept.to.1layhid3 | 3.606448 |
| RPL32.to.1layhid3 | -2.682199 |
| RPS12.to.1layhid3 | 17.1055 |
| RPS14.to.1layhid3 | 0.2339751 |
| RPS23.to.1layhid3 | -7.839087 |
| RPS3.to.1layhid3 | 15.67756 |
| RPS7.to.1layhid3 | -21.78002 |
| SRP9.to.1layhid3 | -12.24064 |
| Intercept.to.1layhid4 | -0.007143841 |
| RPL32.to.1layhid4 | -3.814856 |
| RPS12.to.1layhid4 | 2.777908 |
| RPS14.to.1layhid4 | -2.088647 |
| RPS23.to.1layhid4 | -6.126963 |
| RPS3.to.1layhid4 | -1.531685 |
| RPS7.to.1layhid4 | 11.79065 |
| SRP9.to.1layhid4 | 2.713115 |
| Intercept.to.1layhid5 | -0.882228 |
| RPL32.to.1layhid5 | -5.879788 |
| RPS12.to.1layhid5 | -1.136623 |
| RPS14.to.1layhid5 | 13.89972 |
| RPS23.to.1layhid5 | 6.70285 |
| RPS3.to.1layhid5 | -8.34408 |
| RPS7.to.1layhid5 | 2.809471 |
| SRP9.to.1layhid5 | -8.077643 |
| Intercept.to.1layhid6 | -2.026817 |
| RPL32.to.1layhid6 | -1.444089 |
| RPS12.to.1layhid6 | -8.751555 |
| RPS14.to.1layhid6 | -1.470297 |
| RPS23.to.1layhid6 | 8.654257 |
| RPS3.to.1layhid6 | -6.97356 |
| RPS7.to.1layhid6 | 10.19937 |
| SRP9.to.1layhid6 | 4.284348 |
| Intercept.to.con | 1.622884 |
| 1layhid1.to.con | 4.008299 |
| 1layhid2.to.con | -1.110131 |
| 1layhid3.to.con | -2.266559 |
| 1layhid4.to.con | -3.184323 |
| 1layhid5.to.con | 1.780389 |
| 1layhid6.to.con | -2.421521 |
| Intercept.to.treat | -0.6222848 |
| 1layhid1.to.treat | -4.010048 |
| 1layhid2.to.treat | 1.109789 |
| 1layhid3.to.treat | 2.266677 |
| 1layhid4.to.treat | 3.185193 |
| 1layhid5.to.treat | -1.780552 |
| 1layhid6.to.treat | 2.421188 |
