# Supplementary material for: A signal recognition particle-related joint model of LASSO regression, SVM-RFE and artificial neural network for the diagnosis of systemic sclerosis-associated pulmonary hypertension
Source: Front Genet. 2022 Nov 28;13:1078200. doi: 10.3389/fgene.2022.1078200 (PMC9742487; doi:10.3389/fgene.2022.1078200)
Supplement: Supplementary file 3 [file Table2.DOCX]

**Supplementary Table S2. Code for GSEA based on single gene batch correlation analysis.**

rm(list = ls())

inputFile="" #Read input file

setwd("") #Set up the working directory

data=read.table(inputFile, header=T, sep="\t", check.names=F, row.names=1)

y <- as.numeric(data[1,]) #Move the expression of the gene from the original data to the first row, or change the number in this line of code to the number of the row of the expression of the gene

rownames <- rownames(data)

cor_data_df <- data.frame(rownames)

for (i in 1:length(rownames)){

print(i)

test <- cor.test(as.numeric(data[i,]),y,method="spearman")

cor_data_df[i,2] <- test$estimate

cor_data_df[i,3] <- test$p.value

}

names(cor_data_df) <- c("symbol","correlation","pvalue")

geneList <- cor_data_df$correlation

names(geneList) = cor_data_df$symbol

geneList = sort(geneList, decreasing = TRUE)

library(msigdbr)

library(dplyr)

dd <- msigdbr(species = "Homo sapiens") #Networking required

hallmarks <- dd %>%

filter(gs_cat == "H") %>%

select(gs_name,gene_symbol)

library(clusterProfiler)

y <- GSEA(geneList,TERM2GENE =hallmarks)

library(ggplot2)

dotplot(y,showCategory=12,split=".sign")+facet_grid(~.sign)
