# Supplementary material for: A signal recognition particle-related joint model of LASSO regression, SVM-RFE and artificial neural network for the diagnosis of systemic sclerosis-associated pulmonary hypertension
Source: Front Genet. 2022 Nov 28;13:1078200. doi: 10.3389/fgene.2022.1078200 (PMC9742487; doi:10.3389/fgene.2022.1078200)
Supplement: Supplementary file 5 [file Table3.DOCX]

**Supplementary** **Table S3. Differentially expressed SRP-related genes**

| **Gene** | **Training set** | | **Test set** | |
| --- | --- | --- | --- | --- |
|  | **LogFC** | **P value** | **LogFC** | **P value** |
| RPL10 | -0.067001944 | 0.007287336 | N/A | |
| RPL10A | -0.147151726 | 0.04843367 | -0.1560534 | 0.001456118 |
| RPL10L | -0.094837385 | 0.04167741 | -0.1953537 | 0.03546299 |
| RPL13A | -0.145446479 | 0.01376109 | N/A | |
| RPL15 | -0.115674058 | 0.02732643 | -0.2313663 | 0.001699395 |
| RPL19 | -0.101019216 | 0.04367551 | -0.0905081 | 0.0253595 |
| RPL21 | -0.190342151 | 0.01056425 | N/A | |
| RPL22 | -0.174225832 | 0.002976749 | -0.2548383 | 0.000571777 |
| RPL23 | -0.094894254 | 0.01240447 | N/A | |
| RPL23A | -0.162777543 | 0.000978055 | -0.1071889 | 0.005113141 |
| RPL32 | -0.148508775 | 0.001669725 | -0.1587188 | 0.003679945 |
| RPL35A | -0.117954771 | 0.0476664 | -0.30202 | 0.0002448048 |
| RPL36 | -0.115622843 | 0.04372118 | -0.5177763 | 0.002487539 |
| RPL4 | -0.179048282 | 0.003175841 | N/A | |
| RPL9 | -0.105563465 | 0.03024812 | -0.1250944 | 0.001108055 |
| RPS12 | -0.129455324 | 0.0157761 | -0.1131809 | 0.04749323 |
| RPS14 | -0.117365599 | 0.01995995 | -0.1779221 | 0.000435279 |
| RPS15 | -0.121811718 | 0.04201165 | -0.1323293 | 0.002045005 |
| RPS15A | -0.096737398 | 0.009401645 | -0.1513639 | 0.001645191 |
| RPS18 | -0.139467701 | 0.02778342 | -0.1105628 | 0.01449145 |
| RPS23 | -0.34467686 | 0.04658203 | -0.1074421 | 0.004926894 |
| RPS25 | -0.13588237 | 0.009663031 | -0.1785499 | 0.004480015 |
| RPS3 | -0.155949967 | 0.000347327 | -0.1856216 | 0.000994756 |
| RPS4X | -0.152488181 | 0.02732601 | -0.1403575 | 0.001625154 |
| RPS5 | -0.132032682 | 0.04512041 | -0.3226524 | 0.005058771 |
| RPS6 | -0.117178911 | 0.04198798 | -0.1823227 | 0.00043311 |
| RPS7 | -0.140205037 | 0.002391252 | -0.1340049 | 0.03082895 |
| RPSA | -0.151628319 | 0.00221239 | N/A | |
| SRP9 | -0.070543729 | 0.01301149 | -0.1875652 | 0.03415862 |
| SRPRB | -0.119213102 | 0.02182419 | -0.3011549 | 0.002879473 |
