# Supplementary material for: A signal recognition particle-related joint model of LASSO regression, SVM-RFE and artificial neural network for the diagnosis of systemic sclerosis-associated pulmonary hypertension
Source: Front Genet. 2022 Nov 28;13:1078200. doi: 10.3389/fgene.2022.1078200 (PMC9742487; doi:10.3389/fgene.2022.1078200)
Supplement: Supplementary file 9 [file DataSheet1.DOCX]

Supplementary Material

**A signal recognition particle-related joint model of LASSO regression, SVM-RFE and artificial neural network for the diagnosis of systemic sclerosis-associated pulmonary hypertension**

Jingxi Xu^1,2^, Chaoyang Liang^2^, Jiangtao Li^2*^

^1^North Sichuan Medical College, Nanchong, China

^2^Department of Rheumatology and Immunology, The First People's Hospital of Yibin, Yibin, China

*** Correspondence:**Jiangtao Li
hxyyljt@163.com

# Supplementary Figures and Tables

## Supplementary Figures


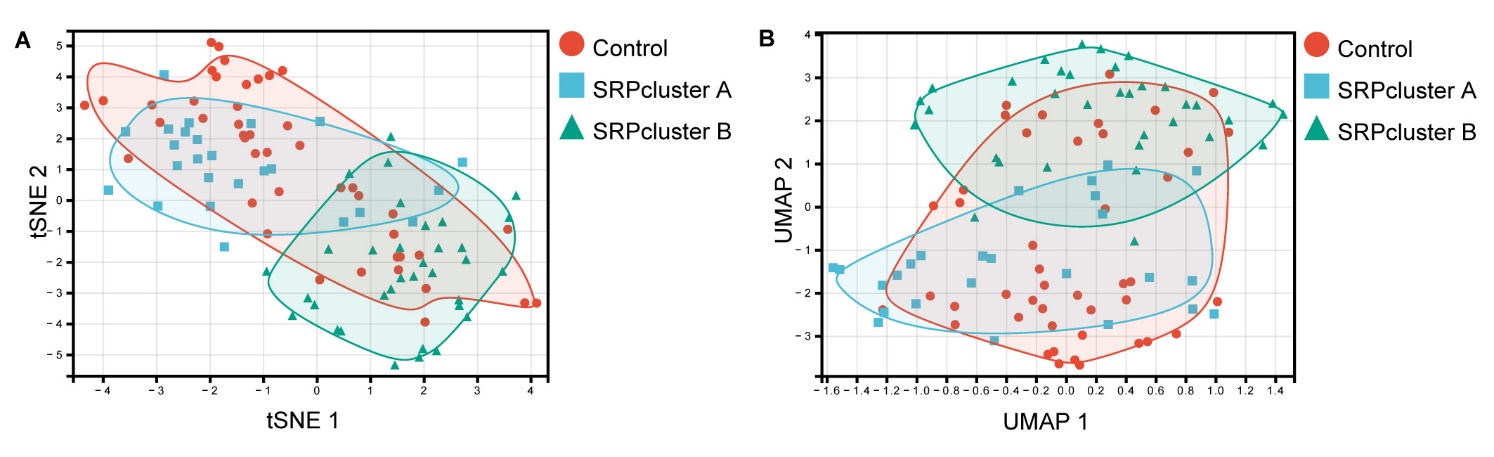


**FIGURE S1** Dimensionality reduction based on 30 DE-SRPGs. **(A)** tSNE plot. **(B)** UMAP plot.


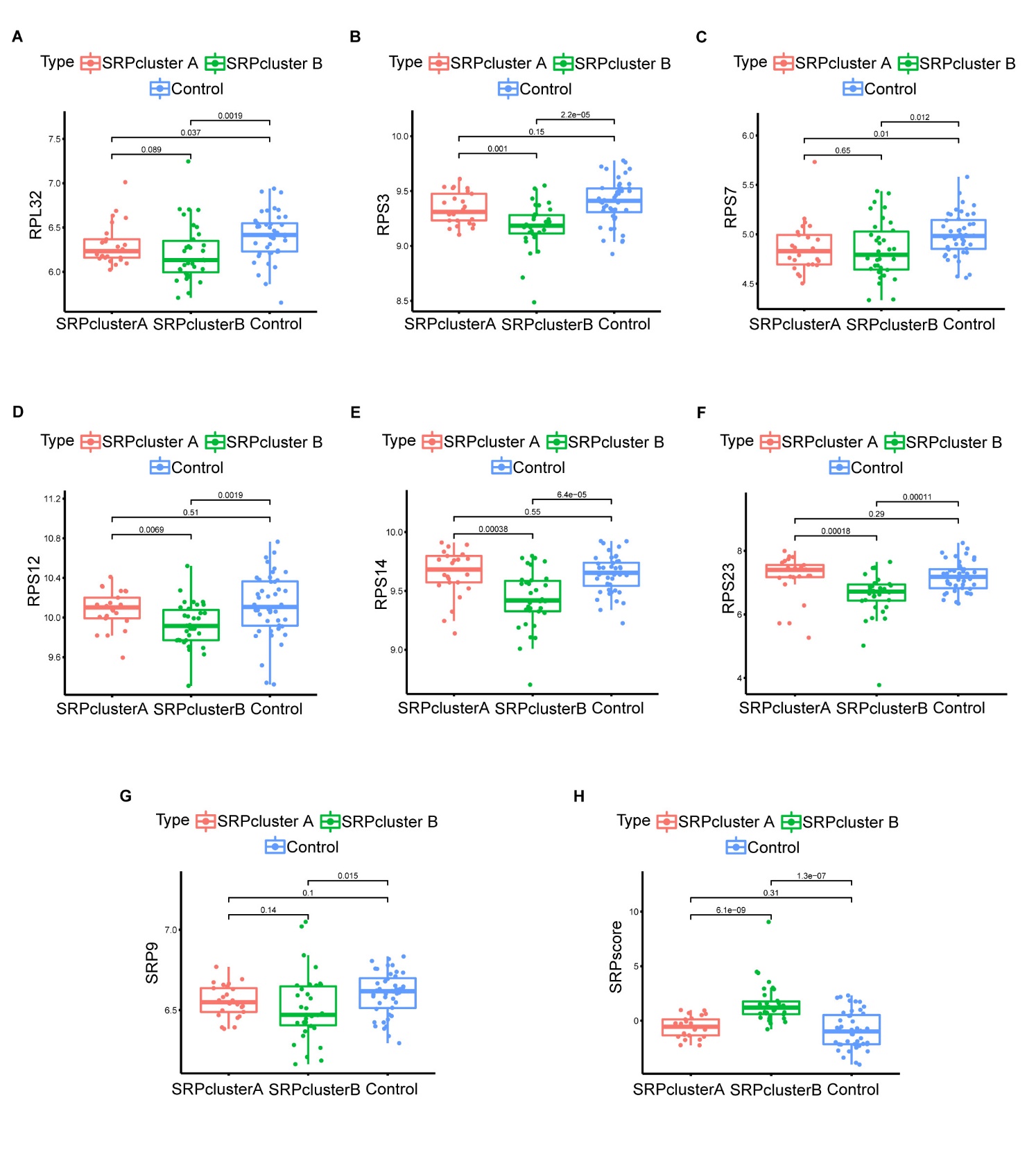


**FIGURE S2** **(A-G)** Expression levels of RPL32, RPS3, RPS7, RPS12, RPS14, RPS23, and SRP9 in SRPcluster A, SRPcluster B, and the control group. **(H)** SRPscore values in SRPcluster A, SRPcluster B and the control group.


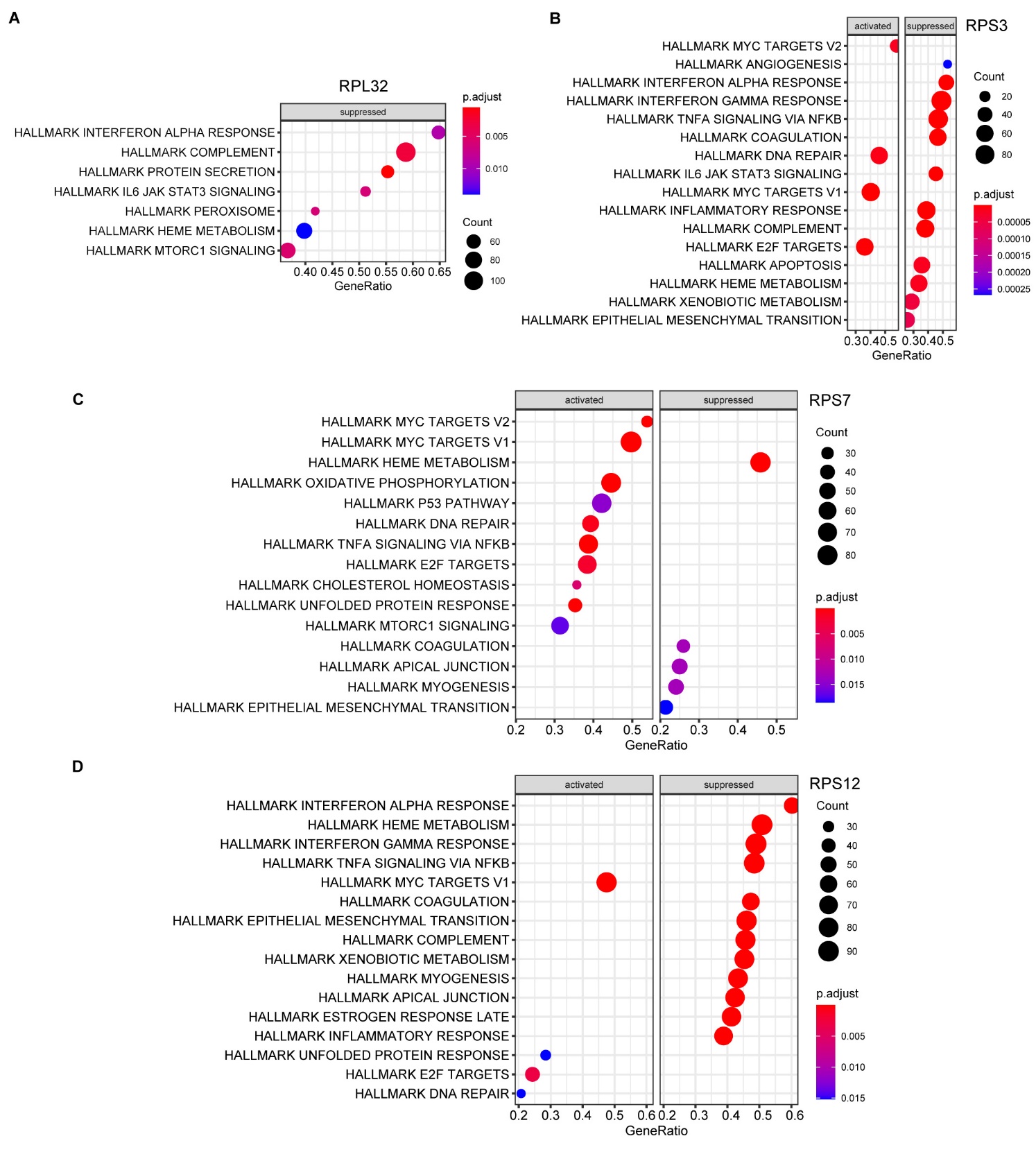


**FIGURE S3 (A-D)** Single gene batch correlation analysis-based GSEA for RPL32, RPS3, RPS7 and RPS12.


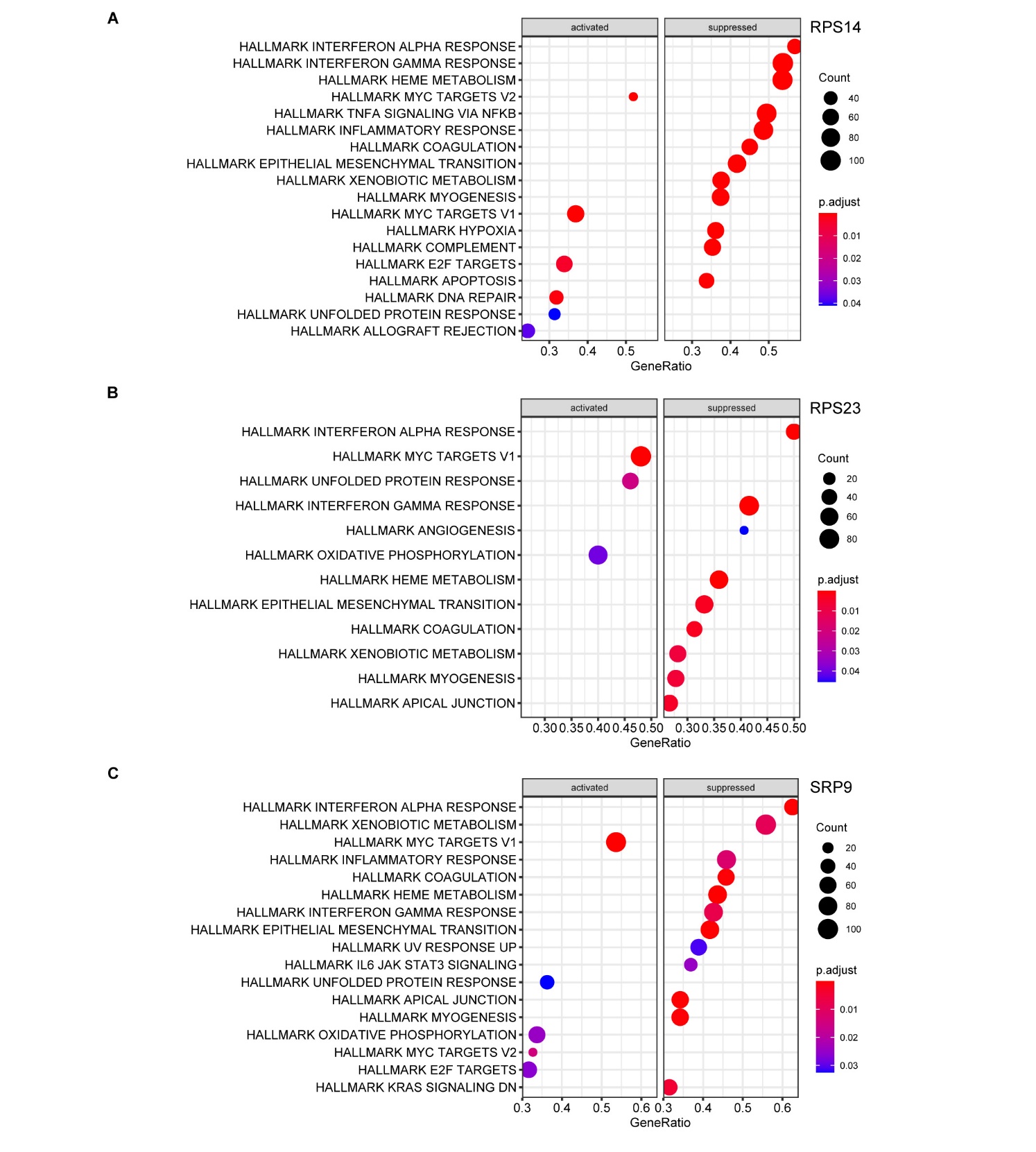


**FIGURE S4 (A-C)** Single gene batch correlation analysis-based GSEA for RPS14, RPS23, and SRP9.

## Supplementary Tables

Supplementary Table S1. The metagenes of 14 SSc-PH-related pathways. (Supplementary Table S1.xlsx)

Supplementary Table S2. Code for GSEA based on single gene batch correlation analysis. (Supplementary Table S2.docx)

Supplementary Table S3. Differentially expressed SRP-related genes. (Supplementary Table S3.docx)

Supplementary Table S4. The GO annotation results of 30 DE-SRPGs. (Supplementary Table S4.xlsx)

Supplementary Table S5. The KEGG enrichment analysis results of 30 DE-SRPGs. (Supplementary Table S5.xlsx)

Supplementary Table S6. The SRPscore values of the samples in the training set. (Supplementary Table S6.xlsx)

Supplementary Table S7. The SRPscore values of the samples in the test set. (Supplementary Table S7.xlsx)

Supplementary Table S8. The output results of the artificial neural network. (Supplementary Table S8.docx)
